# The Influence of Various *N*-Heterocyclic Carbene Ligands on Activity of Nitro-Activated Olefin Metathesis Catalysts

**DOI:** 10.3390/molecules25102282

**Published:** 2020-05-12

**Authors:** Michał Pieczykolan, Justyna Czaban-Jóźwiak, Maura Malinska, Krzysztof Woźniak, Reto Dorta, Anna Rybicka, Anna Kajetanowicz, Karol Grela

**Affiliations:** 1 Institute of Organic Chemistry Polish Academy of Sciences, Kasprzaka 44/52, 01-224 Warsaw, Poland; michalpieczykolan@gmail.com (M.P.); czaban.justyna@gmail.com (J.C.-J.); 2 Faculty of Chemistry, Biological and Chemical Research Centre, University of Warsaw, Żwirki i Wigury 101, 02-089 Warsaw, Poland; mmalinska@chem.uw.edu.pl (M.M.); kwozniak@chem.uw.edu.pl (K.W.); annamariarybicka@gmail.com (A.R.); 3 Department of Chemistry, School of Molecular Sciences, University of Western Australia, 35 Stirling Highway, Perth 6009, Australia; reto.dorta@uwa.edu.au

**Keywords:** metathesis, ruthenium, nitro catalysts, NHC ligands, olefins

## Abstract

A set of nitro-activated ruthenium-based Hoveyda-Grubbs type olefin metathesis catalysts bearing sterically modified *N*-hetero-cyclic carbene (NHC) ligands have been obtained, characterised and studied in a set of model metathesis reactions. It was found that catalysts bearing standard SIMes and SIPr ligands (**4a** and **4b**) gave the best results in metathesis of substrates with more accessible C–C double bonds. At the same time, catalysts bearing engineered naphthyl-substituted NHC ligands (**4d**–**e**) exhibited high activity towards formation of tetrasubstituted C–C double bonds, the reaction which was traditionally Achilles’ heel of the nitro-activated Hoveyda–Grubbs catalyst.

## 1. Introduction

Although first transition metal complexes bearing *N*-heterocyclic carbene (NHC) ligands were studied independently by Wanzlick [1] and Öfele [2] in the late 1960s, these intriguing species remained unexplored for many years. They re-entered the stage in 1991 when Arduengo and co-workers prepared the first stable and crystalline *N*-heterocyclic carbene (IAd) [3]. Since then, because of easy fine-tuning of the steric and electronic properties of these compounds [4], NHCs have been widely used both as organocatalysts and as ligands for numerous transition metals catalysed reactions [5].

**Figure 1:**
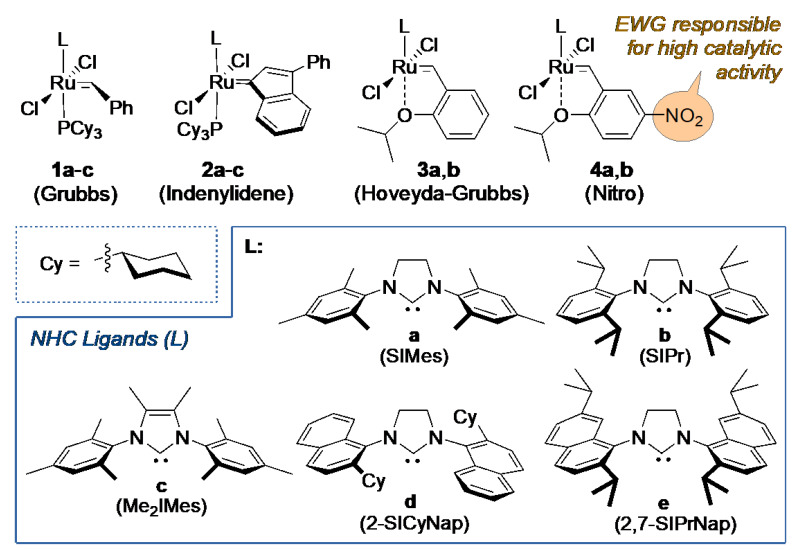
Examples of commercial Ru-based olefin metathesis catalysts and *N*-heterocyclic carbene (NHC) ligands (**a**–**e**).

Olefin metathesis is a useful methodology enabling formation of multiple carbon–carbon double bonds [6,7,8]. Pioneering studies on this reaction were undertaken by scientists working in industry and in academia, where one might mention milestone contributions by Anderson and Merckling (Du Pont–norbornene polymerization) [9], Banks and Bailey (Philips Petroleum—so-called the three-olefin process) [10], and Natta (linear and cyclic olefin polymerization) [11]. In these early contributions, undefined catalytic systems and harsh conditions were usually applied, which limited the applicability of this transformation to rather simple systems. The discovery of Schrock's molybdenum [12] and Grubbs' first-generation ruthenium [13] complexes in the 1990s significantly enhanced pertinence of this methodology, but the real avalanche of olefin metathesis applications happened only after the introduction of the so-called second-generation Ru catalysts, i.e., Ru-complexes bearing at least one NHC ligand [14,15,16]. Currently, a number of complexes are commercially available, inter alia, general-use catalysts like Umicore Grubbs Catalyst M2a (**1a**) [17] introduced in 1999 [18] and its SIPr variant (**1b**), Umicore M2 (**2a**) [19], Hoveyda–Grubbs' catalyst (**3a**) [20] and SIPr analogue (**3b**), and nitro-catalysts **4a**,**b** (Figure 1) [21,22,23].

Given the importance of the NHC ligand in ruthenium olefin metathesis catalysts, these ligands (L) have been optimised over the years. It was found that modification of the central five-membered *N*-heterocycle leads to decreasing activity or faster decomposition of the corresponding complex [24,25]. Similar results were obtained when replacing the aromatic side chain substituents with aliphatic ones [26,27,28,29,30]. However, unsymmetrically substituted NHC ligands, bearing one aromatic and one aliphatic *N*-substituent, have found their important niche as specialised catalysts [31,32,33]. On the other hand, introducing slightly bulkier aryl substituents compared to SIMes [34,35,36,37] or modifications of the 4 and 5 position in the imidazolium ring [26,38,39,40] cause usually an opposite effect resulting in an increase of the catalysts’ activity.

Besides varying the NHC ligand, benzylidene ligands offer a broad testing ground for modifications of the catalytic properties of these ruthenium complexes [41]. Our group has developed a nitro-activated version of the Hoveyda complex **4a** [42,43,44,45]. The presence of an electron-withdrawing group (EWG) [43,46] in *para* position results in weakening of Ru-O bond, therefore accelerating the initiation rate of the resulting catalyst. As a consequence, **4a** has been utilised as a successful metathesis catalyst in natural products and target-oriented syntheses [47,48], as well as the industrial context, such as in the ring-closing metathesis (RCM) at scale up to 7 kg leading to the antiviral BILN 2061 agent precursor at Boehringer–Ingelheim plant [49,50], anticancer agent Largazole at decagrams scale at Oceanyx Pharmaceuticals, Inc. [51], and in continuous flow using a scalable membrane pervaporation device at Snapdragon Chemistry, Inc. [52]. Interestingly, the iodide-containing analogue of **4a** gave very good results in a number of challenging CM and RCM reactions [53]. Importantly, increased stability towards ethylene makes this diiodo derivative especially suitable for macrocyclization RCM of unbiased dienes [53]. Based on the excellent results reported by Bertrand and Grubbs on cyclic-alkyl-amino carbene (CAAC) ligands [54], Skowerski et al. obtained a CAAC analogue of **4a** that promoted difficult RCM macrocyclization at 30 ppm, and cross metathesis of acrylonitrile at 300 ppm Ru loading and lower [55]. In addition, the successful nitro-catalyst design has provided an impetus for developing a number of derivative catalysts utilising the same EWG-activation concept [46,56,57,58]. On the other hand, replacement of the chelating oxygen atom by groups containing sulphur [59,60,61] or nitrogen [61,62,63] results in so-called latent complexes [64,65]. These catalysts exhibit increased stability, but have to be activated thermally, chemically or photochemically.

**Scheme 1:**
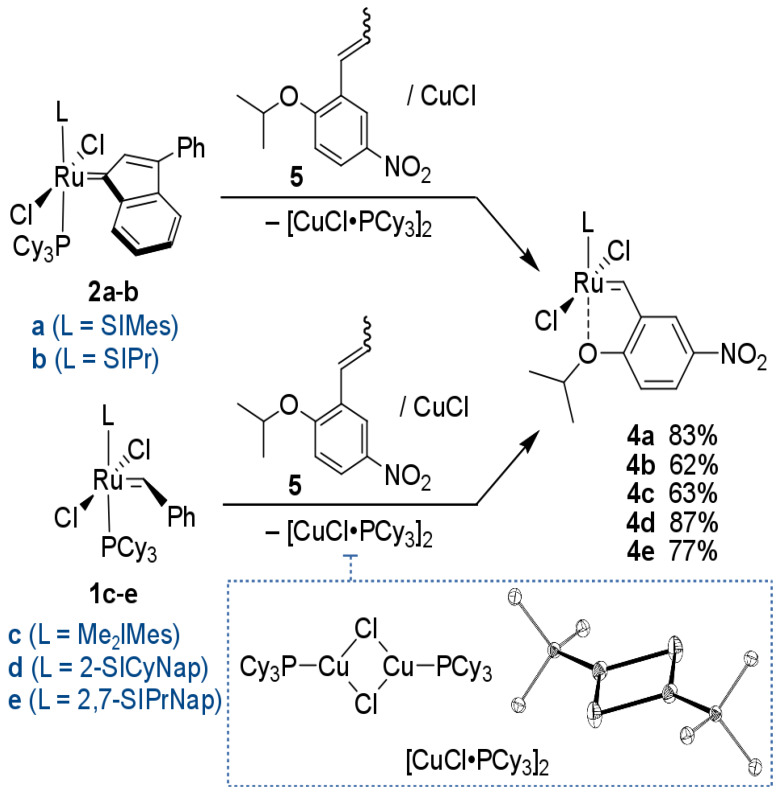
Synthesis of complexes **4a**–**e**.

Herein, we describe the synthesis of a small set of nitro-activated catalysts bearing NHC ligands (L) of different steric properties (Figure 1 and Scheme 1). Catalyst **4a** bearing a well-known SIMes ligand (Figure 1, NHC structures: **a**) was chosen as the benchmark, while the less known SIPr (Figure 1, **4b**) [53] and the new complexes with Me_2_IMes [40] and with two naphthalene based ligands (Figure 1, NHC structures: **c**–**e**) developed by Dorta, were studied in detail [66,67,68,69]. These five complexes were characterised structurally and then tested in model olefin metathesis reactions [70] to check how steric properties of the different NHC ligands influence structural and catalytic properties of the resulting Ru complexes.

## 2. Results and Discussion

### 2.1. Synthesis of the Ruthenium Complexes

All complexes were synthesised via the stoichiometric metathesis-ligand exchange reaction according to a procedure initially disclosed by Hoveyda [71] and illustrated on Scheme 1. Depending on the NHC precursor, the reactions were performed either in toluene or in DCM, in the presence of copper(I) chloride—a commonly used phosphine scavenger [71]. Complexes **4a** and **4b** were obtained from commercially available second-generation indenylidene complexes **2a** and **2b** in 83% and 62% yield, respectively. Interestingly, during these syntheses, we were able to isolate the putative CuCl•PCy_3_ complex in pure form and solve its crystallographic structure. It is stated that despite the fact that CuCl is being used as a phosphine scavenger in the preparation of various Hoveyda complexes for almost 20 years [71], according to our knowledge the product of this reaction has not yet been unambiguously characterised [72]. The synthesis of complexes **4c**–**e** was carried out using appropriate Grubbs second generation complexes **1c**–**e** as the source of ruthenium [40,66,68,73].

Complex **4c** was obtained in the reaction of **1c** with propenylbenzene derivative **5** in the presence of CuCl as a microcrystalline brownish solid with a moderate yield of 63%. Complexes **4d** and **4e** were obtained in a similar way from **5** and corresponding Grubbs-type catalysts [66,68,73] **1d** or **1e** as greenish microcrystalline solids in good yields, 87% and 77% respectively. General conditions for the synthesis of complexes **4a**–**e** are shown in Table 1. As solids, all new Ru-compounds were stable when under an inert atmosphere and were stored for weeks without any sign of decomposition (acc. to TLC and NMR). Having these catalysts in hand, we were ready to study how different NHC arrangements [74] present in **4a**–**e** influence the resulted complex structures and activity.

### 2.2. Structure Analysis

**Figure 2:**
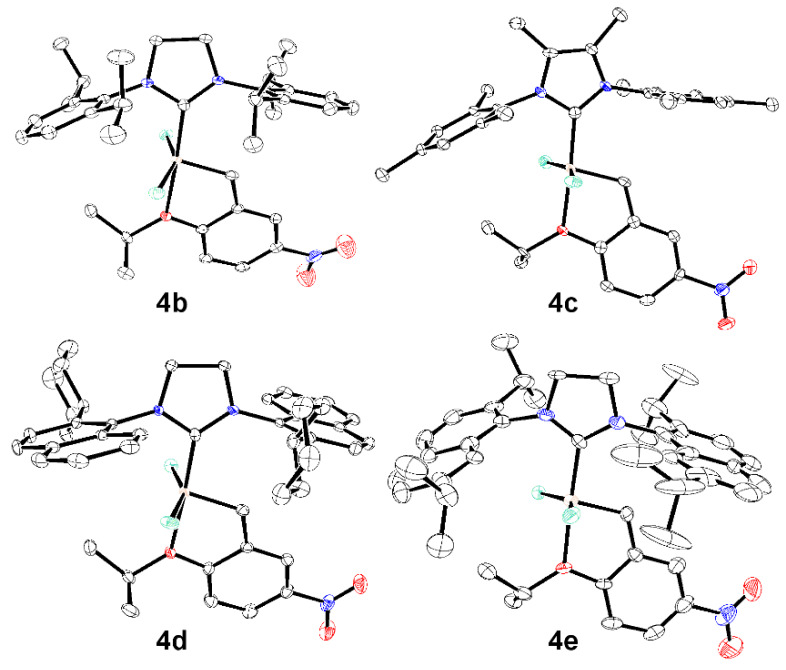
Solid state crystallographic structures of **4a**, **4b**, **4c** and **4e** complexes. Colour codes: blue—nitrogen, red—oxygen, green—chlorine.

**Figure 3:**
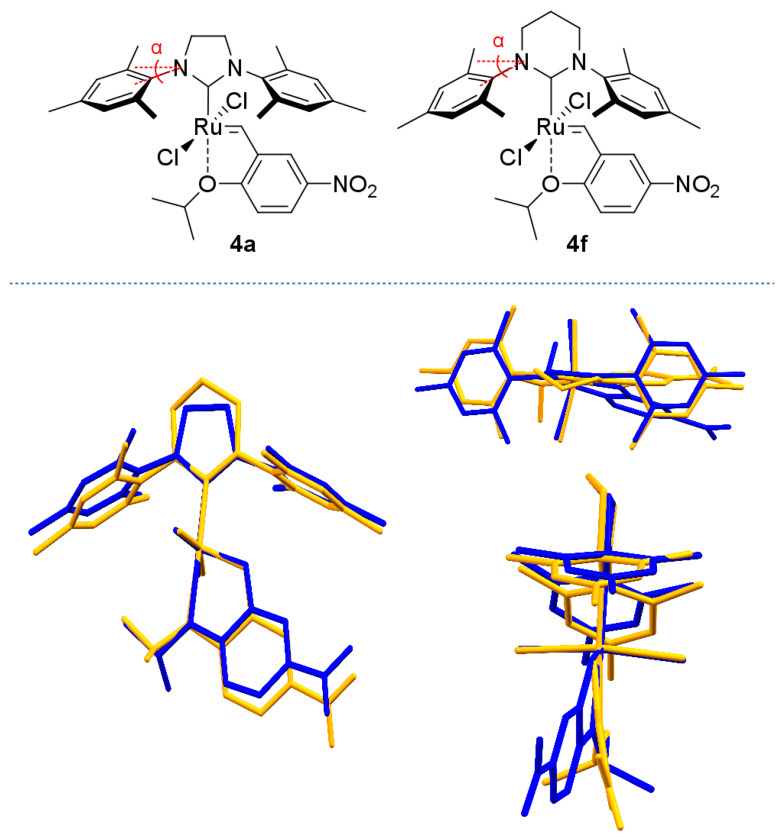
Molecular structures of **4a** and **4f**. Front, top, and side view of molecule overlay of **4a** (blue), **4f** (orange). For angle α values see Table 2.

The crystal structures of **4b**–**e** have been determined by applying single crystal X-ray diffraction (Figure 2). It allows for investigation of structural conformations and steric subtleties of the studied compounds. The structure of **4a** has been previously reported [75] and another related molecule—a catalyst **4f** (Figure 3) developed by Buchmeiser [24] that contains saturated 1,3-*bis*(2,4,6-trimethylphenyl) 3,4,5,6-tetrahydropyrimidin-2-ylidene ligand—was included in Table 2 for comparison purposes [24] (while selected bond lengths and angles are given in Table 2, the full set of X-ray data is provided in Table S1 in Supplementary Materials).

All ruthenium complexes adopt a distorted square bi-pyramid coordination mode around the central ruthenium atom. The top of these pyramids are the O(1) oxygen of the benzylidene chelate and the C(1) carbon atoms of the NHC ligand. The average distance for the Ru-Cl bond amounts to 2.33 Å with a small variation from this value and the chloride atoms are in the trans configuration.

Most of the geometrical parameters do not differ much as they stay in the range of the 3σ threshold, however some interesting trends can be observed. The substitution of various NHC ligands strongly influences the Ru-O(1) bond. The bond is shortened in comparison to the parent SIMes-bearing (**4a**) compound (2.287(1) Å) with the exception of the NHC ring modification to the 6-member one in the **4f** moiety (2.310(2) Å).

An opposite trend was found for the Ru-C(1) bond, which is elongated except for the **4d** molecule. The Ru-C(2) bond changes within a smaller range with the shortest distance for the **4a** and **4c** (1.825(2)Å and 1.821(3) Å, respectively), whereas the longest bond distance is recorded for the **4e** structure 1.836(9) Å and 1.838(9) Å).

**Figure 4:**
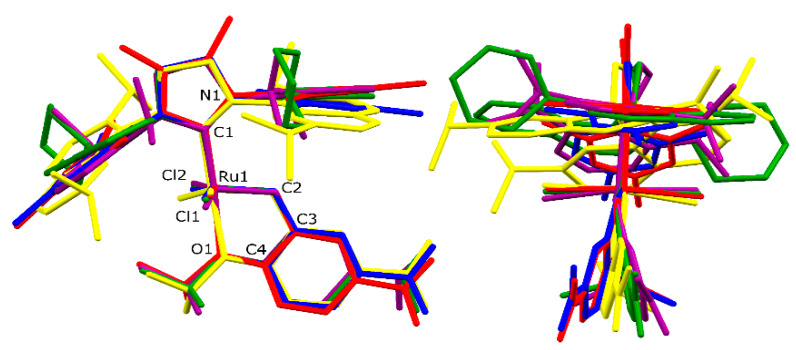
Front and side view of molecule overlay of **4a** (blue), **4b** (purple), **4c** (red), **4d** (green) and **4e** (yellow) complexes with label used to define selected geometrical parameters.

The torsion angles are the most sensitive parameters in the crystal studies, and they differ in all studied complexes, although not that significantly. The Ru-C(2)-C(3)-C(4)-O(1) ring in the Hoveyda pre-catalyst is almost planar. The Ru-C(2)-C(3)-C(4) torsion angle, which defines the mutual orientation of the carbene bond and the NHC ligand is more flexible (Figure 4). Yet again, similar values were found for complexes **4a** and **4c** that form negative torsion angles, whereas one can see positive values of this angle for the **4b** and **4d** complexes, with the **4e** precatalyst in the middle of the range. The ligands with more bulky character demand a bigger rotation of the C(2)-Ru-C(1)-N(1) torsion angle (**4b**, **4d** and **4e**).

The angle α (Figure 3) represents visually how much the *N*-aryl ‘wings’ of the NHC ligands are lowered towards the metal centre. For (S)IMes-decorated complexes (**4a**, **4c**) this angle measures 19.8 and 20.1°, and is only slightly larger (20.8°) in the case of SIPr bearing **4b**. Importantly, the naphthyl members of the series have the *N*-substituents more ‘up’ (19.0° for **4d**), being in strong contrast to complex **4f** where the NHC wings are visibly lowered (25.4°) thus shielding the Ru centre more. However, some individual geometrical parameters can mislead the overall comparison. The root means square (RMS) analysis, taking into account the following six atoms: Ru, C(1), C(2), O(1), Cl(1) and Cl(2), has revealed similarity of the studied structures to the initial **4a** compound. The RMS values are 0.090, 0.053, 0.057, 0.049, 0.062 Å and 0.104 Å for **4b**, **4c**, **4d**, **4e′**, **4e′′** and **4f**, respectively. The structures **4c**, **4d** and **4e′** revealed a bigger similarity to the **4a** complex, and this finding agrees with the V_bur%_ values.

**Figure 5:**
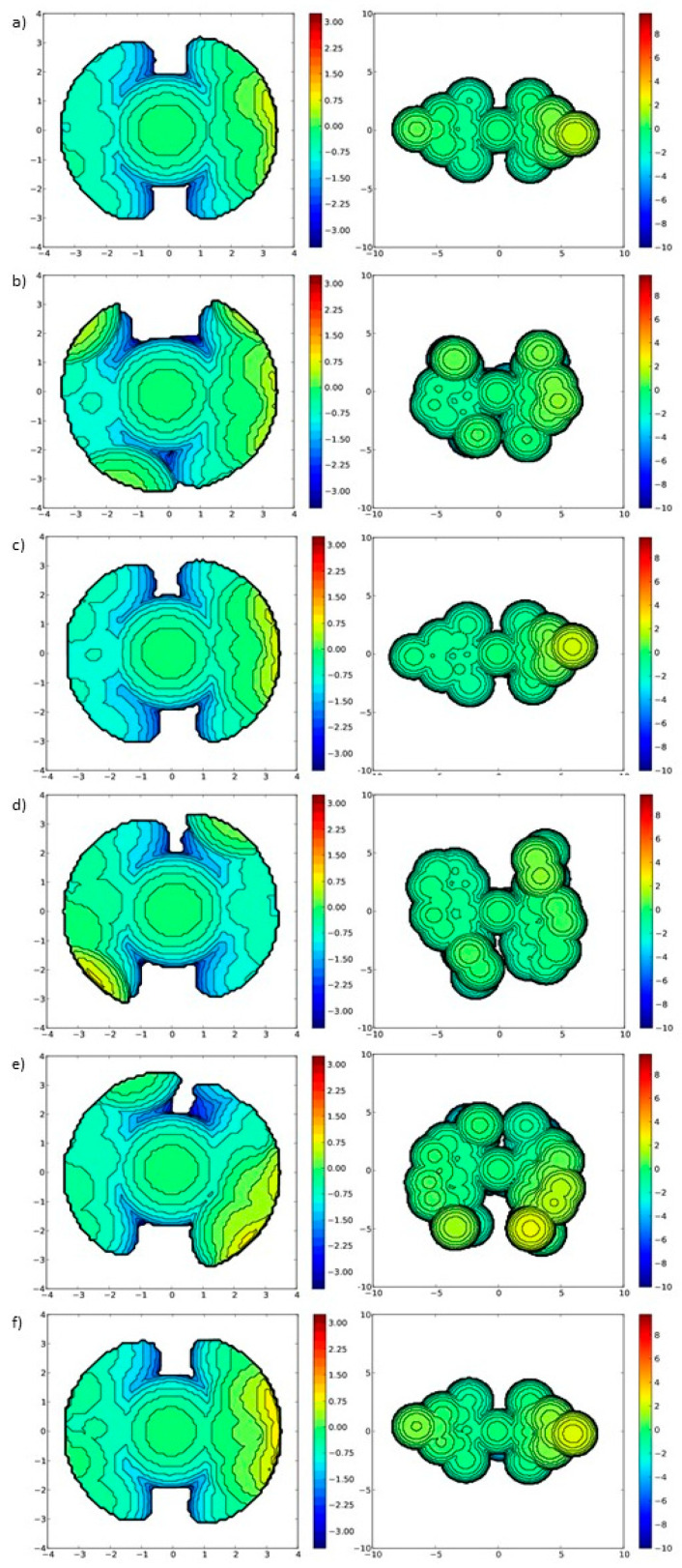
Steric maps calculated in SambVca software for complexes **4a** to **4f**. Standard (3.5 Å) and enlarged (10 Å) radii were used. (**a**) Catalyst **4a**, (**b**) Catalyst **4b**, (**c**) Catalyst **4c**, (**d**) Catalyst **4d**, (**e**) Catalyst **4e**, (**f**) Catalyst **4f**.

Using the data obtained from diffraction studies, we also calculated the buried volume (V_bur%_) parameters [76] for the studied series of nitro-catalysts bearing NHC ligands (Figure 5). As expected, V_bur%_ value of SIPr in **4b** (36.5%) was bigger than the one of SIMes in **4a** (35.4%) and Me_2_IMes in **4c** (34.7%). The value obtained for Dorta's 2-SICyNap, present in catalyst **4d** (34.6%) was similar to the one obtained for Me_2_IMes in **4c** (34.7%), even though the (cyclohexyl)naphthyl groups in **4d** can be considered as relatively bulkier in comparison with smaller Mes *N*-substituents in **4c**. Therefore it seems that they have similar steric demand of ligand (at least in the proximity of Ru).

Because crystals of catalyst **4e** that were measured by us contained two molecules in the asymmetric unit (**4e′** and **4e′′**), the V_bur%_ values were calculated for each of them (Table 2). The relatively big difference between them was probably caused by various spatial arrangements of the naphthyl groups in both of these molecules. **4a** and **4c** had the smallest NHC, while the **4d** and **4e′′** the largest. In the case of compounds **4b** and **4e"** the greater steric hindrance around the Ru atom is visible on the V_bur_ maps, which directly influenced the increased calculated V_bur_ value. The least protected Ru centre in this series is visible for complexes **4c** and **4d** and corresponds to the lowest V_bur_ values. Buchmeiser's catalyst **4f** has the highest V_bur_ value, which is probably correlated to the presence of the pyrimidine ring and different electron density than for the other NHCs. The difference between **4f** and other catalysts is marginal and is best visible for 10 Å radii (Figure 5f), where below the central atom some negative electron density is visible.

### 2.3. Comparative Catalytic Activity Studies of Nitro-Catalysts **4a**–**e**

**Scheme 2:**
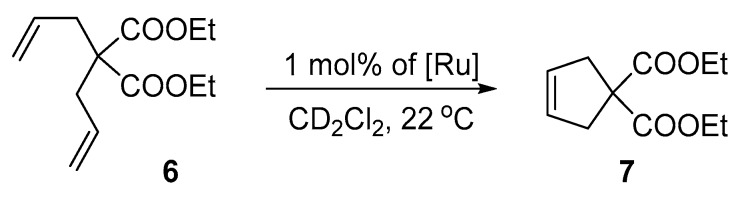
Ring-closing metathesis (RCM) of diethyl 2,2-diallylmalonate (**6**).

The performance of the nitro-catalysts **4a**–**e** was evaluated in the ring-closing and ene-yne metathesis of six model substrates: diethyl 2,2-diallylmalonate (**6**), diethyl 2-allyl-2-(2-methylallyl)malonate (**8**), 2,2-di(2-methylallyl)tosylate (**10**), diethyl 2,2-di(2-methylallyl)malonate (**12**), allyl 1,1-diphenylpropargyl ether (**14**), and (1-(prop-1-en-2-yl-methoxy)prop-2-yne-1,1-diyl)dibenzene (**16**). In the first stage of this research the RCM reaction of 2,2-diallylmalonate (**6**), the most commonly used model substrate [77] was examined in the presence of 1 mol% of nitro-catalysts **4a**–**e** (Scheme 2). Reactions were performed in NMR tubes at ambient temperature.

**Figure 6:**
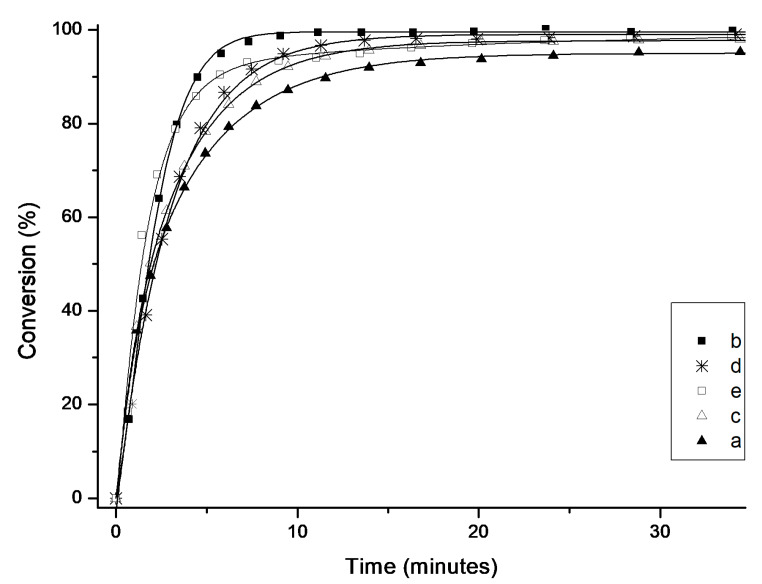
Time/conversion curves for the RCM reaction of diethyl 2,2-diallylmalonate (**6**) with 1 mol% of **4a**–**e** at 23 °C (monitored by ^1^H-NMR). Lines are visual aids only.

As expected for the EWG-activated family of catalysts, all tested complexes showed high activity and almost quantitative conversion was achieved after only 10–15 min (Figure 6).

Interestingly, the least active complex was the SIMes-containing **4a**. Complex **4c** with an unsaturated Me_2_IMes ligand initiated slightly faster and provided maximal conversion after approximately 15 minutes. Both catalysts containing naphthyl substituents in their NHC ligands **4d** and **4e** exhibited even higher activity, with a slightly better result obtained for cyclohexyl-substituted catalyst **4d**. The most active complex in the series was **4b** bearing the SIPr ligand, which gave full conversion in less than 10 min.

**Scheme 3:**
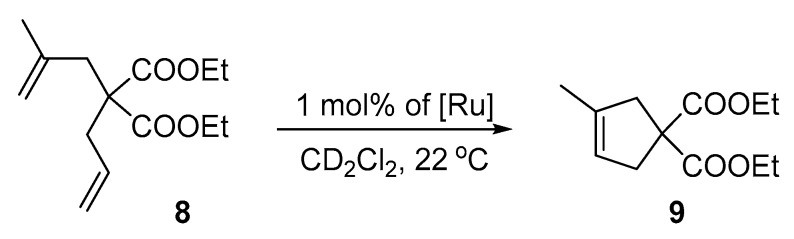
RCM reaction of diethyl 2-allyl-2-(2-methylallyl)malonate (**8**).

**Figure 7:**
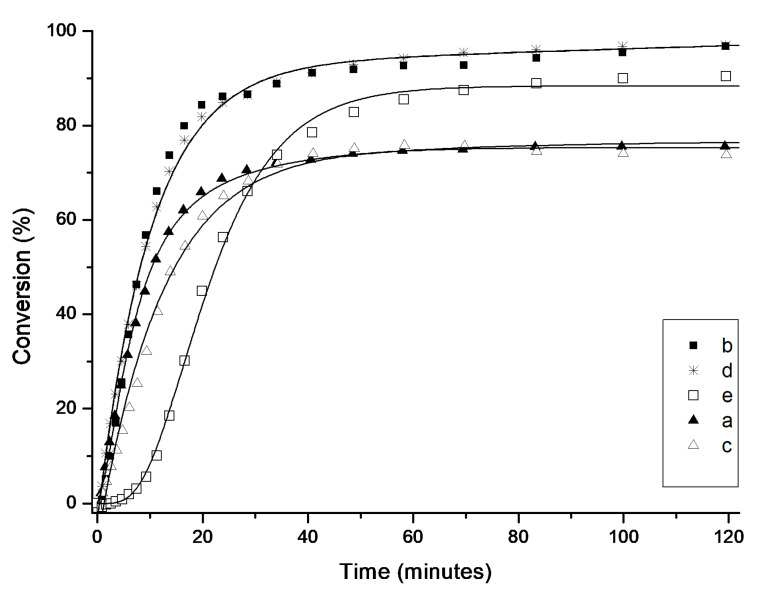
Time/conversion curves for the RCM reaction of diethyl 2-allyl-2-(2-methylallyl)malonate (**8**) with 1 mol% of **4a**–**e** at 23 °C (monitored by ^1^H-NMR). Lines are visual aids only.

Diethyl 2,2-diallylmalonate (**6**) is a rather simple substrate to ring-close and is used more to examine whether a newly obtained complex exhibits any catalytic metathesis activity than to show in detail the subtle differences between similarly active catalysts. To determine how the activity of the new Ru-complexes compare, a more difficult substrate containing substituted double bonds, diethyl 2-allyl-2-(2-methyllyl)malonate (**8**) was studied next (Scheme 3, Figure 7).

In this case, under similarly mild conditions as used for the RCM of **6**, namely in the presence of 1 mol% catalyst and at room temperature, the general trend was maintained, although higher diversity between the tested catalysts was found (Figure 7). The highest activity was observed for **4b** (SIPr) and **4d**, (2-SICyNap) complexes containing relatively large aryl substituents in the NHC ligand. After 20 minutes they reached 84 and 81% yield, respectively, reaching in both cases 97% of RCM product **9** within 2 h.

An interesting S-shaped curve was observed for the second complex bearing bulky naphthyl-substituted NHC (**4e**). The latter admittedly initialised slower than the other counterparts, as after 20 min it reached only 45% conversion. Interestingly, after this initial latency period **4e** initiated in a fast rate giving after 100 min 90% conversion of **8**. As observed previously for the conversion of **6** to **7**, the least reactive complexes in this model reaction were **4a** and **4c**, which initiated quicker than **4e** but after two hours provided the product with only 75 and 73% yield, respectively.

**Scheme 4:**
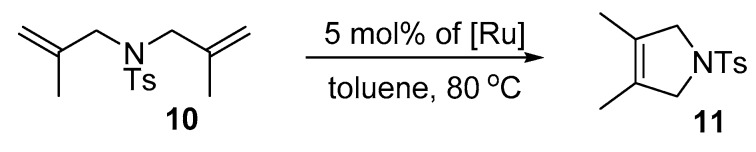
RCM reaction of 2,2-di(2-methylallyl)tosylate (**10**).

Next, we examined the activity of the nitro-catalysts in the RCM formation of tetrasubstituted olefins. Dienes 2,2-di(2-methyl allyl)tosylate (**10**) and diethyl 2,2-di(2-methyl allyl)malonate (**12**) are known to be more demanding and usually require the use of harsh conditions and specifically designed catalysts in order to achieve high yields [78]. Here, the reactions were performed in the presence of 5 mol% of complexes **4a**–**e** at 80 °C (Scheme 4).

**Figure 8:**
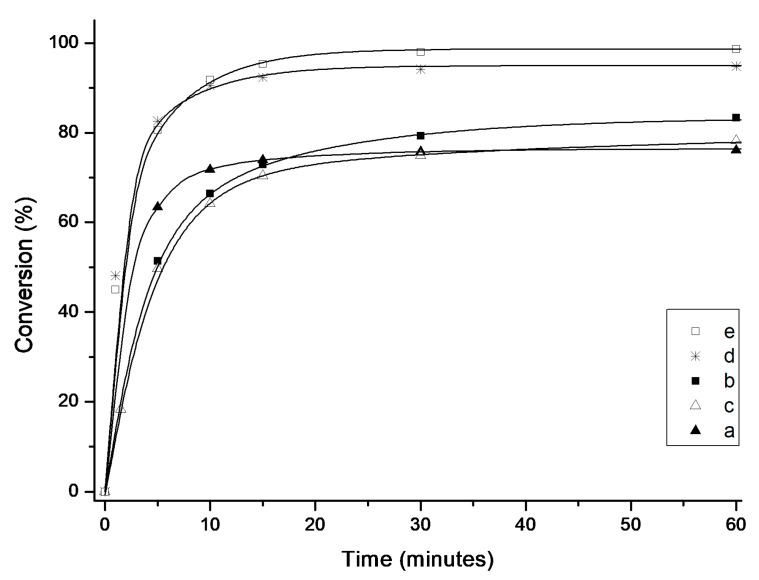
Time/conversion curves for the RCM reaction of 2,2-di(2-methylallyl)tosylate (**10**) with 5 mol% of **4a**–**e** at 80 °C (monitored by GC). Lines are visual aids only.

When tosylate **10** was used (Scheme 4), good to very good yields were achieved, however a significant difference between complexes bearing bulky naphthyl substituted NHCs (**4d** and **4e**) and the more standard ones (**4a**–**c**) was observed (Figure 8). Despite the forcing conditions such as high catalyst loading and elevated temperature, the SIMes, SIPr and Me_2_IMes-bearing catalysts produced **10** in 76–83% yield after one hour. In contrast, bulkier Dorta-type complexes **4d** and **4e** reached almost quantitative conversion, 90 and 91% respectively, after only 20 min. Interestingly, when the reaction time was extended to 24 h also complex with SIPr ligand (**4b**) achieved a similar conversion of 91%, while **4a** and **4c** died reaching 80–82% only (Table 3).

**Scheme 5:**
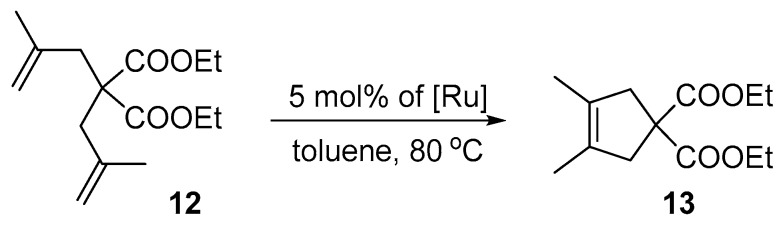
RCM reaction of diethyl 2,2-di(2-methylallyl)malonate (**12**).

When even more challenging diethyl 2,2-di(2-methylallyl)malonate (**12**) was used instead of 2,2-di(2-methylallyl)tosylate (**10**) in the presence of 5 mol% of Ru at 80 °C (Scheme 5, Table 4), only the most bulky Dorta complex **4e** provided a relatively satisfactory result of 62% yield in 6 h. The other complexes (**4a**–**d**) were less active, leading to 9–21% of the desired product **13**.

After extending the reaction time to 24 h, virtually no changes in conversion were observed in the case of complexes with mesitylene-based NHC ligands (**4a** and **4c**). Pleasurably, we noticed a huge improvement in yield of the desired product when the reaction was conducted in the presence of Dorta-type (**4e** and **4d**) and SIPr-based (**4b**) catalysts (Table 4). Especially in the case of the latter two, changes were significant (from 14% to 66% for **4d** and from 9% to 63% for **4b**).

**Scheme 6:**
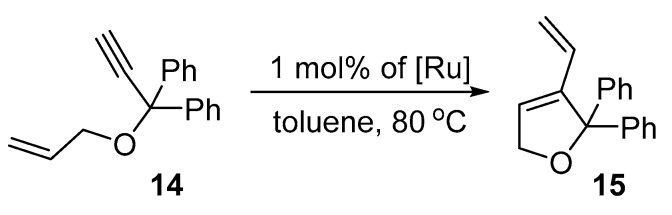
Ene-yne reaction of allyl 1,1-diphenylpropargyl ether (**14**).

Ene-yne metathesis is a highly selective and atom-economical methodology for the synthesis of 1,3 dienes, which are valuable building blocks in organic synthesis [79]. To picture the application profile of the studied catalysts, two members of the ene-yne class of compounds were investigated with catalysts **4a**–**e**. Reactivity profiles for the metathesis of allyl 1,1-diphenylpropargyl ether (**14**) were established first (Scheme 6).

**Figure 9:**
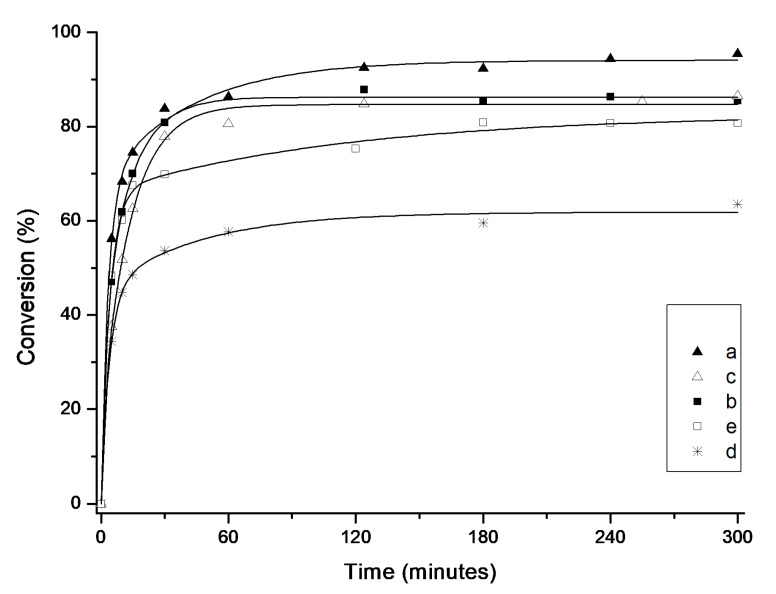
Time/conversion curves for the ene-yne reaction of allyl 1,1-diphenylpropargyl ether (**14**) with 1 mol% of **4a**–**e** at 80 °C (monitored by GC). Lines are visual aids only.

In the ene-yne cycloisomerisation of easy to react **14** [80] the most active were catalysts containing the smallest substituents (**4a**–**c**), while those with more bulky side chain groups (**4d**–**e**) showed diminished conversions (Figure 9). Nevertheless, all catalysts, but one, **4d** contain a large cyclohexyl substituent in the *ortho* position of the aryl ring, provided the desired product with yields above 80% during the first 6 h of the reaction. Further extension of the reaction time to 24 h resulted in slight improvement of the results leading to essentially quantitative conversions (over 90%) for **4a** and **4c** (Table 5).

**Scheme 7:**
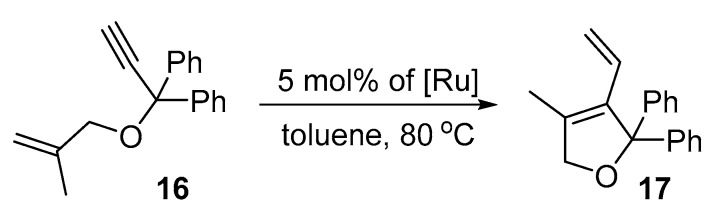
Ene-yne reaction of (1-(prop-1-en-2-yl-methoxy)prop-2-yne-1,1-diyl)dibenzene (**16**).

Next, the more challenging cycloisomerisation substrate (1-(prop-1-en-2-yl-methoxy)prop-2-yne-1,1-diyl)dibenzene (**16**) [81,82,83] was utilised (Scheme 7). As for substrate **12**, also in this case the loading of the catalysts was increased from 1 to 5 mol% in order to obtain near-quantitative conversions.

Indeed, most of the complexes used gave the expected product with a yield of 90–100% in less than 6 hours, with **4d** being the most active, and after extension of the reaction time to 24 h 100% of yield was reached (Table 6). The only exception was the complex **4b** containing simple SIPr-ligand, which under these conditions gave only 25 and 41% conversion, after 6 and 24 h respectively.

## 3. Experimental Section

### 3.1. General

All reactions were carried out under argon flow in pre-dried glassware using Schlenk techniques. Reaction profiles performed in NMR tube were carried out in degassed CD_2_Cl_2_. CH_2_Cl_2_ (Sigma-Aldrich Sp. z o.o., Poznan, Poland) was dried by distillation with CaH_2_ under argon and was stored under argon. THF, toluene, *n*-hexane and xylene were dried by distillation with Na/K alloy. Flash chromatography was performed using Merck KGaA (Darmstadt, Germany) silica gel 60 (230–400 mesh). NMR spectra were recorded in CDCl_3_ or CD_2_Cl_2_ with Varian Mercury 400 MHz and Varian VNMRS 500 MHz spectrometers. MS (FD/FAB) was recorded with a GCT Premier spectrometer from Waters Corporation (Milford, MA, USA). MS (EI) spectra were recorded with an AMD 604 Intectra GmbH (Harpstedt, Germany) spectrometer. Other commercially available chemicals were used as received.

### 3.2. Synthesis of Complexes

**Synthesis of 4a**: Complex (**2a**) (220 mg, 0.232 mmol) was dissolved in toluene (7 mL), and 1-isopropoxy-4-nitro-2-(prop-1-en-1-yl)benzene (**5**) (61.6 mg, 0.278 mmol) was added. The mixture was stirred for 5 min, CuCl (45.9 mg, 0.474 mmol) was added, and the mixture was heated at 80 °C for 30 min. The reaction mixture was cooled to room temperature and concentrated in vacuo. From this point, all manipulations were carried out in air with reagent grade solvents. The product was purified by silica gel chromatography (AcOEt/c-hexane = 1:4 *v*/*v*). The solvent was evaporated under vacuum, and the residue was dissolved in CH_2_Cl_2_ (2 mL). MeOH (5 mL) was added and CH_2_Cl_2_ was slowly removed under vacuum. The precipitated was filtered, washed with MeOH (5 mL), and dried in vacuo to afford **4a** as a green microcrystalline solid (130 mg, 83%). ^1^H-NMR (CD_2_Cl_2_, 500 MHz,): δ = 16.42 (s, 1H), 8.46 (dd, *J* = 9.1, 2.5 Hz, 1H), 7.80 (d, *J* = 2.5 Hz, 1H), 7.10 (s, 4H), 6.94 (d, *J* = 9.1 Hz, 1H), 5.01 (sept, *J* = 6.1 Hz, 1H), 4.22 (s, 4H), 2.46–2.48 (m, 18H), 1.30 (d, *J* = 6.1 Hz, 6H); ^13^C-NMR (125 MHz, CD_2_Cl_2_): δ = 289.1, 208.2, 156.8, 150.3, 145.0, 143.5, 139.6, 139.3, 129.8, 124.5, 117.2, 113.3, 78.2, 52.0, 21.3, 21.2, 19.4; IR (KBr): $\tilde{v}$ = 2924, 2850, 1606, 1521, 1480, 1262, 1093, 918, 745 cm^−1^; FDMS *m*/*z* [M^+^] 671.1.

**Synthesis of 4b**: Similar to the preparation of **4a**, **5** (150 mg, 0.68 mmol) was added to the solution of complex **2b** (690 mg, 0.68 mmol) in toluene (15 mL). The mixture was stirred for 5 min, and CuCl (135 mg, 1.36 mmol) was added. **4b** was obtained as green microcrystalline solid (380 mg, 62%). ^1^H-NMR (500 MHz, CD_2_Cl_2_): δ = 16.33 (s, 1H), 8.38 (dd, *J* = 9.0, 2.7 Hz, 1H), 7.69 (d, *J* = 2.7 Hz, 1H), 7.58 (t, *J*= 7.7 Hz, 4H), 7.39 (d, *J* = 7.7 Hz, 4H), 6.90 (d, *J* = 9.0 Hz, 1H), 4.99 (m, 1H), 4.20 (s, 4H), 3.56 (m, 4H), 1.40 (d, *J* = 6.1 Hz, 6H), 1.24 (d, *J* = 6.7 Hz, 12H); ^13^C-NMR (125 MHz, CD_2_Cl_2_): δ = 283.7, 210.3, 156.6, 149.0, 143.6, 143.0, 136.2, 130.0, 124.4, 124.0, 116.7, 112.8, 77.7, 77.2, 77.0, 76.7, 54.5, 28.8, 26.5, 23.3, 21.7; IR (KBr):$\tilde{v}$= 3096, 3069, 2970, 2951, 2927, 2868, 1527, 1341, 1270, 1095, 914, 742 cm^−1^; FDMS *m*/*z* [M^+^] 755.10.

**Synthesis 4c**: Complex **2c** (500 mg, 0.571 mmol) was dissolved in toluene (11 mL), and 1-isopropoxy-4-nitro-2-(prop-1-en-1-yl)benzene (**5**) (190 mg, 0.856 mmol) was added. The mixture was stirred for 5 min, CuCl (113 mg, 1.14 mmol) was added, and the mixture was stirred at 70 °C for 40 min. The reaction mixture was cooled to room temperature and concentrated in vacuo. From this point, all manipulations were carried out in air with reagent grade solvents. The product was purified by silica gel chromatography (AcOEt/c-hexane = 1:5 *v*/*v*). The solvent was evaporated under vacuum, and the residue was dissolved in CH_2_Cl_2_ (2 mL). MeOH (5 mL) was added and CH_2_Cl_2_ was slowly removed under vacuum. The precipitated was filtered, washed with MeOH (5 mL) and dried in vacuo to afford **4c** as a brownish microcrystalline solid (250 mg, 63%). ^1^H-NMR (500 MHz, CD_2_Cl_2_): δ = 16.57 (s, 1H), 8.42 (dd, *J* = 9.0, 2.7 Hz, 1H), 7.92 (d, *J* = 2.5 Hz, 1H), 7.14 (s, 4H), 6.89 (d, *J* = 9.0 Hz, 1H), 4.98 (m, 1H), 2.48 (s, 6H), 2.18 (s, 12H), 1.97 (s, 6H), 1.35 (d, *J* = 6.1 Hz, 6H); ^13^C-NMR (125 MHz, CD_2_Cl_2_): δ = 287.0, 167.0, 156.4, 145.0, 143.2, 139.7, 138.4, 129.2, 127.8, 123.3, 116.7, 112.7, 77.5, 77.3, 77.0, 76.8, 21.8, 21.1, 21.1, 19.1, 19.1; IR (KBr): $\tilde{v}$ = 3103, 3084, 2986, 2969, 2921, 1604, 1571, 1520, 1384, 1337, 1320, 1095, 746, 660 cm^−1^; FDMS *m*/*z* [M^+^] 697.1.

**Synthesis of 4d**: Similar to the preparation of **4c**, **5** (86 mg, 0.389 mmol) was added to the solution of complex **1d** (250 mg, 0.243 mmol) in CH_2_Cl_2_ (15 mL). The mixture was stirred for 5 min, and CuCl (48 mg, 0.486 mmol) was added. **4d** was obtained as green microcrystalline solid (180 mg, 87 %). ^1^H-NMR (500 MHz, CD_2_Cl_2_): δ = 16.04 (s, 1H), 8.30 (d, *J* = 8.1 Hz, 2H), 8.23 (dd, *J* = 9.0, 2.7 Hz, 1H), 8.08 (d *J* = 8.6, 2H), 7.95 (d, *J* = 7.9 Hz, 2H), 7.68 (d, *J* = 8.6 Hz, 2H), 7.60 (td, *J* = 6.9, 1.0 Hz, 2H), 7.52 (td, *J* = 6.9, 1.0 Hz, 2H), 7.33 (d *J* = 2.5, 1H), 6.71 (d, *J* = 9.0 Hz, 1H), 4.77 (m, 1H), 4.48–4.34 (m, 4H), 4.12 (q, *J* = 14.2, 7.1 Hz, 1H), 3.11 (s, 2H), 2.16 (s, 1H), 2.04 (s, 2H), 1.98–1.96 (m, 12H), 1.74–1.55 (m, 10H), 1.48–1.37 (m, 4H), 1.25 (t, *J* = 7.1 Hz, 4H), 1.09 (d, *J* = 6.1 Hz, 2H), 1.01 (d, *J* = 6.1 Hz, 2H); ^13^C-NMR (125 MHz, CD_2_Cl_2_): δ = 211.5, 156.5, 145.1, 143.7, 142.8, 133.0, 131.6, 129.8, 127.9, 127.0, 126.2, 125.2, 123.9, 116.6, 112.4, 77.5, 77.2, 77.0, 76.7, 60.3, 54.5, 53.4, 39.8, 36.2, 32.5, 31.5, 30.9, 28.2, 27.5, 26.6, 26.3, 25.8, 21.1; IR (KBr): $\tilde{v}$ = 3067, 2925, 2849, 1735, 1523, 1441, 1340, 1267, 1091, 914, 818, 747 cm^−1^; FDMS *m*/*z* [M^+^] 851.2.

**Synthesis of 4e**: Similar to the preparation of **4c**, **5** (64.4 mg, 0.291 mmol) was added to the solution of complex **1e** (200 mg, 0.194 mmol) in CH_2_Cl_2_ (10 mL). The mixture was stirred for 5 min, and CuCl (38.4 mg, 0.388 mmol) was added. **4e** was obtained as green microcrystalline solid (128 mg, 77 %). ^1^H-NMR (500 MHz, CD_2_Cl_2_): δ = 16.39 (s, 1H), 16.21 (s, 1H), 8.21 (dq, *J* = 8.9, 2.5 Hz, 1H), 8.05 (d, *J* = 8.9 Hz, 2H), 7.95 (s, 1H), 7.86 (dd *J* = 8.3, 3.2, 2H), 7.63 (t, *J* = 8.9 Hz, 2H), 7.50 (d, *J* = 2.5 Hz, 1H), 7.47–7.40 (m, 2H), 6.69 (t, *J* = 8.3 Hz, 1H), 4.80–4.70 (m 1H), 4.60 (s, 1H), 4.47 (t, *J* = 5.8 Hz, 2H), 3.65 (quint, *J* = 13.1, 6.7 Hz, 1H), 3.22 (quint, *J* = 13.5, 6.7 Hz, 1H), 3.11 (quint, *J* = 13.5, 6.8 Hz, 1H), 1.44–1.36 (m, 25H), 1.13 (d, *J* = 5.9 2H), 1.04 (d, *J* = 6.0 Hz, 2H), 0.93 (d, *J* = 6.0 Hz, 2H); ^13^C-NMR (125 MHz, CD_2_Cl_2_): δ = 286.9, 286.6, 211.2, 210.7, 156.5, 147.0, 146.1, 143.9, 143.8, 142.9, 131.7, 131.0, 129.8, 129.7, 127.7, 126.3, 125.5, 123.7, 123.3, 123.2, 122.4, 116.6, 112.5, 112.4, 77.4, 77.2, 77.0, 76.7, 54.0, 34.7, 34.4, 29.2, 29.1, 25.8, 24.0, 23.5, 23.5, 23.3, 22.8, 22.6, 21.1, 21.0, 20.7; IR (KBr): $\tilde{v}$ = 3090, 3058, 2960, 2870, 1604, 1525, 1473, 1340, 1256, 1092, 845 cm^−1^; FDMS *m*/*z* [M^+^] 855.3.

## 4. Conclusions

The family of nitro-complexes containing NHC ligands with different steric properties was synthesised, characterised and investigated in terms of activity. Analysis of the solid-state geometrical parameters manifested some interesting relationships. Intuitively, the most important difference in geometry was expressed by angle α, representing visually how the *N*-aryl ‘wings’ of the NHC ligand are lowered towards the metal centre (Figure 4, Table 2). In the case of the SIPr-bearing **4b** the *N*-aryl ‘flaps’ are slightly lowered compared to (S)IMes-decorated **4a**,**c**. Interestingly, the naphthyl members of this series (**4d**–**e**) have the *N*-substituents even slightly more ‘elevated’ compared to their (S)IMes and SIPr counterparts (**4a**–**b**). This is in strong contrast to complex **4f** where the NHC wings are visibly lowered, thus shielding much more the Ru centre. Interestingly, the latter complex, although very useful in cyclopolymerization of diynes, in model RCM reactions was found to be less reactive than the analogue SIMes Hoveyda-Grubbs complex [24]. The V_bur%_ values and steric maps calculated for the studied complexes illustrated the same picture, rendering the naphthyl-containing complexes **4d**–**e** being the least crowded and **4f** having the highest V_bur%_ value. The model reactions also sorted the tested complexes into two groups. While in the reaction of a simple model diene (**6**) all catalysts exhibited similarly high activity, in the case of a still rather straightforward cycloisomerisation of ene-yne **14**, the less bulky NHC containing complexes **4a**–**c** were more active. At the same time, with more demanding sterically crowded substrates, a significant advantage of complexes with bulkier NHC ligands (**4b**, **4d**–**e**) was evident. Importantly, complexes **4d**–**e** demonstrated high activity in formation of tetra-substituted C–C double bonds [78,84], the reaction which was traditionally Achilles’ heel of the nitro-catalyst [42,43,44,45]. It is stressed that in all cases the studied model reactions were very clean and no side-products were observed.

Overall, the comparative study here suggests that the elaborated naphthyl-based catalysts (**4d**–**e**) may be better for challenging, sterically crowded substrates, while the ‘easy’ substrates can be transformed more readily in the presence of catalysts with standard NHC ligands (**4a**–**b**). Interestingly, catalysts **4c** bearing Me_2_IMes ligand seemed the least utile.

These results show again [85] that different catalysts can be optimal for different applications, and that even small, sometimes incremental, variations can result in substantial changes in reactivity.

## Figures and Tables

**Table 1:** Detailed conditions used in synthesis of **4a**–**e**.

Catalyst	Solvent	Time (min)	Temp. (°C)	Yield (%)
**4a**	Toluene	60	80	83
**4b**	Toluene	60	80	60
**4c**	Toluene	60	60	63
**4d**	DCM	20	40	87
**4e**	DCM	10	40	77

**Table 2:** Selected bond lengths (Å) and angles (deg) in complexes **4a**–**e**. The **4e** structure contains two molecules in the asymmetric unit.

	4a [75]	4b	4c	4d	4e ^a^	4f [24]
Ru‒C(1)	1.979(3)	1.985(4)	1.990(4)	1.970(3)	2.002(9) 2.012(9)	2.013(2)
Ru‒O(1)	2.287(1)	2.244(2)	2.232(2)	2.254(2)	2.285(5) 2.252(5)	2.310(2)
Ru‒C(2)	1.825(2)	1.829(4)	1.821(3)	1.827(3)	1.836(9) 1.838(9)	1.825(3)
Ru‒Cl(1)	2.333(1)	2.324(1)	2.335(1)	2.328(1)	2.328(2) 2.328(2)	2.343(1)
Ru‒Cl(2)	2.330(1)	2.333(1)	2.339(1)	2.331(1)	2.328(2) 2.324(2)	2.343(1)
C(1)-Ru-O(1)	178.45(6)	172.5(1)	175.6(1)	175.4(1)	176.8(3) 177.8(3)	175.93(8)
C(1)-Ru-C(2)	101.36(8)	102.1(1)	103.0(1)	102.4(1)	99.9(4) 101.6(4)	105.1(1)
Ru-C(2)-C(3)-C(4)	8.6(2)	−8.9(5)	5.9(4)	−5.4(3)	−2(1) 2(1)	−4.5(3)
C(2)-Ru-C(1)-N(1)	7.6(2)	14.2(4)	5.5(4)	13.0(3)	−31.8(9) 27(1)	0.8(2)
α	19.8(1)	20.8(3)	20.1(2)	19.0(2)	19.4(7) 12.4(7)	25.4(2)
V_Bur_ (%)	35.4	36.5	34.7	34.6	34.8 36.1	38.0

^a^ The **4e** structure contains two molecules in the asymmetric unit.

**Table 3:** RCM reaction of 2,2-di(2-methylallyl)tosylate (**10**) with 5 mol% of **4a**–**e** at 80 °C (monitored by GC) after 1 and 24 h.

Catalyst	Conversion (%)	
	After 1 h	After 24 h
**4a**	76	82
**4b**	83	91
**4c**	78	80
**4d**	98	99
**4e**	95	97

**Table 4:** RCM reaction of diethyl 2,2-di(2-methylallyl)malonate (**12**) with 5 mol% **of 4a**–**e** at 80 °C (monitored by GC) after 6 and 24 h.

Catalyst	Conversion (%)	
	After 6 h	After 24 h
**4a**	16	18
**4b**	9	63
**4c**	21	21
**4d**	14	66
**4e**	62	84

**Table 5:** Ene-yne reaction of allyl 1,1-diphenylpropargyl ether (**14**) with 1 mol% of **4a**–**e** at 80 °C (monitored by GC) after 6 and 24 h.

Catalyst	Conversion (%)	
	After 6 h	After 24 h
**4a**	93	99
**4b**	87	87
**4c**	90	93
**4d**	60	73
**4e**	81	88

**Table 6:** Ene-yne reaction of (1-(prop-1-en-2-yl-methoxy)prop-2-yne-1,1-diyl)dibenzene (**16**) with 5 mol% of **4a**–**e** at 80 °C (monitored by GC) after 6 and 24 h.

Catalyst	Conversion (%)	
	After 6 h	After 24 h
**4a**	88	99
**4b**	15	42
**4c**	94	97
**4d**	94	98
**4e**	74	98

## Supplementary Materials

The following are available online. Figure S1: Atomic Displacement Parameters (ADPs) and the labeling of atoms in **4b** and **4c**; Figure S2: Atomic Displacement Parameters (ADPs) and the labeling of atoms in **4d**; Figure S3: Atomic Displacement Parameters (ADPs) and the labeling of atoms in 4e for two molecules in asymmetric unit (**4e′** and **4e′′**); Figure S4: Overlay of molecules from the **4a** structure (black) with the **4b** (magenta), **4c** structure (blue), **4d** structure (green), **4f** structure (grey), **4e′** structure (red) and **4e′′** (yellow) and **4g** (grey; Figure S5: Atomic Displacement Parameters; Table S1: Experimental details for **4b–4e** structures; Table S2: Experimental details for the CuClPCy3 measurement.
