# Supplementary material for: The Influence of Various N-Heterocyclic Carbene Ligands on Activity of Nitro-Activated Olefin Metathesis Catalysts
Source: Molecules. 2020 May 12;25(10):2282. doi: 10.3390/molecules25102282 (PMC7287594; doi:10.3390/molecules25102282)
Supplement: Supplementary file 1 [file molecules-25-02282-s001.pdf]

SUPPORTING INFORMARION

The influence of various N-heterocyclic carbene ligands on activity of nitro-activated olefin metathesis catalyst

Michał Pieczykolan [1,2], Justyna Czaban [1], Maura Malinska [2], Krzysztof Woźniak [2], Reto Dorta [3], Anna Rybicka [2], Anna Kajetanowicz [2,*], and Karol Grela [1,2,*]

1 Institute of Organic Chemistry Polish Academy of Sciences, Kasprzaka 44/52, 01-224 Warsaw, Poland

2 Faculty of Chemistry, Biological and Chemical Research Centre, University of Warsaw, Żwirki i Wigury 101, 02-089 Warsaw, Poland

3 Department of Chemistry, School of Molecular Sciences, University of Western Australia, 35 Stirling Highway, 6009 Perth, Australia

E-mail: karol.grela@gmail.com, a.kajetanowicz@uw.edu.pl

General Remarks 3
Synthesis of ruthenium complexes 3
  Synthesis of **4a** 3
  Synthesis of **4b** 4
  Synthesis of **4c** 4
  Synthesis of **4d** 5
  Synthesis of **4e** 5
Model metathesis reactions 6
  General comparative RCM (NMR measurements) 6
    Diethyl cyclopent-3-ene-1,1-dicarboxlate (**7**) 6
    Diethyl 3-methylcyclopent-3-ene-1,1-dicarboxylate (**9**) 6
  Comparative RCM experiments (GC measurements) 6
    3,4-Dimethyl-1-tosyl-2,5-dihydro-1*H*-pyrrole (**11**) 7

Diethyl 3,4-dimethylcyclopent-3-ene-1,1-dicarboxylate (**13**) 7
Comparative ene-yne experiments (GC measurements) 7
2,2-Diphenyl-3-vinyl-2,5-dihydro-furan (**15**) 7
4-Methyl-2,2-diphenyl-3-vinyl-2,5-dihydro-furan (**17**) 7
X-Ray crystallography 8
NMR spectra 15
References 19

General Remarks

All air and moisture sensitive reactions were carried out under argon atmosphere in pre-dried glassware using Schlenk techniques. The catalyst preparation was carried out under argon in pre-dried glassware using Schlenk techniques. The anhydrous solvents were dried by distillation over the following drying agents and were transferred under argon: THF (over sodium benzophenone ketyl and distilled under argon), toluene (Na), n-pentane, n-hexane, n-pentane, CH_2Cl_2 (CaH_2). Flash column chromatography was performed using Merck silica gel 60 (230-400 mesh). Analytical thin-layer chromatography (TLC) of all reactions was performed on silica gel 60 F254 TLC plates. Visualization was performed with standard potassium permanganate stains or UV light. NMR spectra were recorded in CD_2Cl_2; using Varian Mercury 400 MHz, Varian VNMRS 500 MHz and Varian VNMRS 600 MHz spectrometers; chemical shifts (δ) are given in ppm relative to TMS, coupling constants are (J) in Hz. Multiplicities are abbreviated as follows: singlet (s), doublet (d), triplet (t), quartet (q), quintet (quint), septet (sept), multiplet (m), and broad (br). IR spectra: wavenumbers are in cm^{-1}. MS (FD/FAB) were recorded on GCT Premier spectrometer from Waters. MS (EI, LSIMS) spectra were recorded on AMD 604 Intectra GmbH spectrometer. MS (ESI) spectra were recorded on Mariner Perseptive Biosystems, Inc. GC/MS measurements were done on HP 5890 with HP 5 column. Micro-analyses were provided by Institute of Organic Chemistry, PAS, Warsaw. Model substrates were obtained according to literature procedures.[1] All commercial chemicals were used without further purification.

Synthesis of ruthenium complexes

Synthesis of 4a

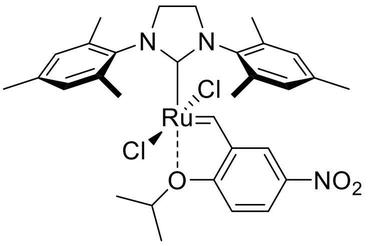

Complex **2a** (220 mg, 0.232 mmol) was dissolved in toluene (7 mL), and 1-isopropoxy-4-nitro-2-(prop-1-en-1-yl)benzene (**5**) (61.6 mg, 0.278 mmol) was added. The mixture was stirred for 5 min, CuCl (45.9 mg, 0.474 mmol) was added, and the mixture was heated at 80 °C for 30 minutes. The reaction mixture was cooled to room temperature and concentrated *in vacuo*. From this point, all manipulations were carried out in air with reagent grade solvents. The product was purified by silica gel chromatography (AcOEt/c-hexane = 1:4 v/v). The solvent was evaporated under vacuum, and the residue was dissolved in DCM (2 mL). MeOH (5 mL) was added and DCM was slowly removed under vacuum. The precipitated was filtered, washed with MeOH (5 mL), and dried in *vacuo* to afford **4a** as a green microcrystalline solid (130 mg, 83%). ^1H NMR (CD_2Cl_2, 500 MHz,): δ = 16.42 (s, 1H), 8.46 (dd, *J* = 9.1, 2.5 Hz, 1H), 7.80 (d, *J* = 2.5 Hz, 1H), 7.10 (s, 4H), 6.94 (d, *J* = 9.1 Hz, 1H), 5.01 (sept, *J* = 6.1 Hz, 1H), 4.22 (s, 4H), 2.46-

2.48 (m, 18H), 1.30 (d, *J* = 6.1 Hz, 6H); ^{13}C NMR (125 MHz, CD_2Cl_2): δ = 289.1, 208.2, 156.8, 150.3, 145.0, 143.5, 139.6, 139.3, 129.8, 124.5, 117.2, 113.3, 78.2, 52.0, 21.3, 21.2, 19.4; IR (KBr): ṽ = 2924, 2850, 1606, 1521, 1480, 1262, 1093, 918, 745 cm^{-1}; FDMS *m/z* [M+·] 671.1.

Synthesis of 4b

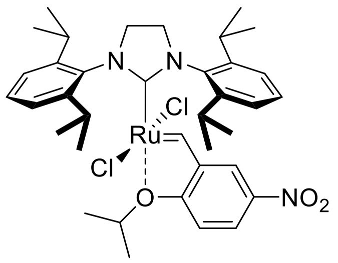

Similar to the preparation of **4a**, **5** (150 mg, 0.68 mmol) was added to solution of complex **2b** (690 mg, 0.68 mmol) in toluene (15 mL). The mixture was stirred for 5 min, and CuCl (135 mg, 1.36 mmol) was added. **4b** was obtained as green microcrystalline solid (380 mg, 62%). ^{1}H NMR (500 MHz, CD_2Cl_2): δ = 16.33 (s, 1H), 8.38 (dd, *J* = 9.0, 2.7 Hz, 1H), 7.69 (d, *J* = 2.7 Hz, 1H), 7.58 (t, *J* = 7.7 Hz, 4H), 7.39 (d, *J* = 7.7 Hz, 4H), 6.90 (d, *J* = 9.0 Hz, 1H), 4.99 (m, 1H), 4.20 (s, 4H), 3.56 (m, 4H), 1.40 (d, *J* = 6.1 Hz, 6H), 1.24 (d, *J* = 6.7 Hz, 12H); ^{13}C NMR (125 MHz, CD_2Cl_2): δ = 283.7, 210.3, 156.6, 149.0, 143.6, 143.0, 136.2, 130.0, 124.4, 124.0, 116.7, 112.8, 77.7, 77.2, 77.0, 76.7, 54.5, 28.8, 26.5, 23.3, 21.7; IR (KBr): ṽ = 3096, 3069, 2970, 2951, 2927, 2868, 1527, 1341, 1270, 1095, 914, 742 cm^{-1}; FDMS *m/z* [M+·] 755.1.

Synthesis of 4c

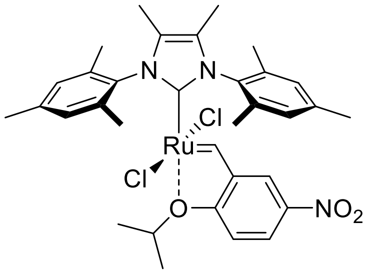

Complex **2c** (500 mg, 0.571 mmol) was dissolved in toluene (11 mL), and 1-isopropoxy-4-nitro-2-(prop-1-en-1-yl)benzene (**5**) (190 mg, 0.856 mmol) was added. The mixture was stirred for 5 min, CuCl (113 mg, 1.14 mmol) was added, and the mixture was stirred at 70 °C for 40 minutes. The reaction mixture was cooled to room temperature and concentrated in *vacuo*. From this point, all manipulations were carried out in air with reagent grade solvents. The product was purified by silica gel chromatography (AcOEt/*c*-hexane = 1:5 v/v). The solvent was evaporated under vacuum, and the residue was dissolved in DCM (2 mL). MeOH (5 mL) was added and DCM was slowly removed under vacuum. The precipitated was filtered, washed with MeOH (5 mL), and dried in *vacuo* to afford **4c** as a brownish microcrystalline solid (250 mg, 63%). ^{1}H NMR (500 MHz, CD_2Cl_2): δ = 16.57 (s, 1H), 8.42 (dd, *J* = 9.0, 2.7 Hz, 1H), 7.92 (d, *J* = 2.5 Hz, 1H), 7.14 (s, 4H), 6.89 (d, *J* = 9.0 Hz, 1H), 4.98 (m, 1H), 2.48 (s, 6H), 2.18 (s, 12H), 1.97 (s, 6H), 1.35 (d, *J* = 6.1 Hz, 6H); ^{13}C NMR (125 MHz, CD_2Cl_2): δ = 287.0, 167.0, 156.4, 145.0, 143.2, 139.7, 138.4, 129.2, 127.8, 123.3, 116.7, 112.7, 77.5, 77.3, 77.0, 76.8, 21.8, 21.1, 21.1, 19.1, 19.1; IR (KBr): ṽ = 3103, 3084,

2986, 2969, 2921, 1604, 1571, 1520, 1384, 1337, 1320, 1095, 746, 660 cm^{-1}; FDMS *m/z* [M+·] 697.1.

Synthesis of 4d

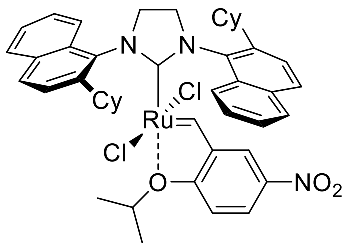

Similar to the preparation of **4c**, **5** (86 mg, 0.389 mmol) was added to solution of complex **1d** (250 mg, 0.243 mmol) in DCM (15 mL). The mixture was stirred for 5 min, and CuCl (48 mg, 0.486 mmol) was added. **4d** was obtained as green microcrystalline solid (180 mg, 87%). ^{1}H NMR (500 MHz, CD_2Cl_2): δ = 16.04 (s, 1H), 8.30 (d, *J* = 8.1 Hz, 2H), 8.23 (dd, *J* = 9.0, 2.7 Hz, 1H), 8.08 (d, *J* = 8.6, 2H), 7.95 (d, *J* = 7.9 Hz, 2H), 7.68 (d, *J* = 8.6 Hz, 2H), 7.60 (td, *J* = 6.9, 1.0 Hz, 2H), 7.52 (td, *J* = 6.9, 1.0 Hz, 2H), 7.33 (d, *J* = 2.5, 1H), 6.71 (d, *J* = 9.0 Hz, 1H), 4.77 (m, 1H), 4.48-4.34 (m, 4H), 4.12 (q, *J* = 14.2, 7.1 Hz, 1H), 3.11 (s, 2H), 2.16 (s, 1H), 2.04 (s, 2H), 1.98-1.96 (m, 12H), 1.74-1.55 (m, 10H), 1.48-1.37 (m, 4H), 1.25 (t, *J* = 7.1 Hz, 4H), 1.09 (d, *J* = 6.1 Hz, 2H), 1.01 (d, *J* = 6.1 Hz, 2H); ^{13}C NMR (125 MHz, CD_2Cl_2): δ = 211.5, 156.5, 145.1, 143.7, 142.8, 133.0, 131.6, 129.8, 127.9, 127.0, 126.2, 125.2, 123.9, 116.6, 112.4, 77.5, 77.2, 77.0, 76.7, 60.3, 54.5, 53.4, 39.8, 36.2, 32.5, 31.5, 30.9, 28.2, 27.5, 26.6, 26.3, 25.8, 21.1; IR (KBr): ṽ = 3067, 2925, 2849, 1735, 1523, 1441, 1340, 1267, 1091, 914, 818, 747 cm^{-1}; FDMS *m/z* [M+·] 851.2.

Synthesis of 4e

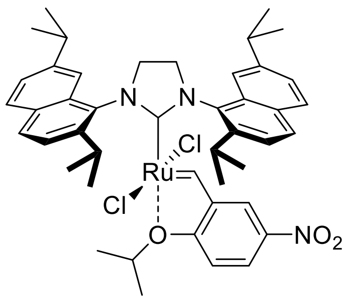

Similar to the preparation of **4c**, **5** (64.4 mg, 0.291 mmol) was added to solution of complex **1e** (200 mg, 0.194 mmol) in DCM (10 mL). The mixture was stirred for 5 min, and CuCl (38.4 mg, 0.388 mmol) was added. **4e** was obtained as green microcrystalline solid (128 mg, 77 %). ^{1}H NMR (500 MHz, CD_2Cl_2): δ = 16.39 (s, 1H), 16.21 (s, 1H), 8.21 (dq, *J* = 8.9, 2.5 Hz, 1H), 8.05 (d, *J* = 8.9 Hz, 2H), 7.95 (s, 1H), 7.86 (dd *J* = 8.3, 3.2, 2H), 7.63 (t, *J* = 8.9 Hz, 2H), 7.50 (d, *J* = 2.5 Hz, 1H), 7.47-7.40 (m, 2H), 6.69 (t, *J* = 8.3 Hz, 1H), 4.80-4.70 (m 1H), 4.60 (s, 1H), 4.47 (t, *J* = 5.8 Hz, 2H), 3.65 (quint, *J* = 13.1, 6.7 Hz, 1H), 3.22 (quint, *J* = 13.5, 6.7 Hz, 1H), 3.11 (quint, *J* = 13.5, 6.8 Hz, 1H), 1.44-1.36 (m, 25H), 1.13 (d, *J* = 5.9 2H), 1.04 (d, *J* = 6.0 Hz, 2H), 0.93 (d, *J* = 6.0 Hz, 2H); ^{13}C NMR (125 MHz, CD_2Cl_2): δ = 286.9, 286.6, 211.2, 210.7, 156.5, 147.0, 146.1, 143.9, 143.8, 142.9, 131.7, 131.0, 129.8, 129.7, 127.7, 126.3, 125.5, 123.7, 123.3, 123.2, 122.4, 116.6, 112.5, 112.4, 77.4, 77.2, 77.0, 76.7, 54.0, 34.7, 34.4, 29.2, 29.1, 25.8, 24.0, 23.5, 23.5, 23.3, 22.8, 22.6, 21.1, 21.0, 20.7; IR (KBr): ṽ = 3090, 3058, 2960, 2870, 1604, 1525, 1473, 1340, 1256, 1092, 845 cm^{-1}; FDMS *m/z* [M^{+}] 855.3.

Model metathesis reactions

General comparative RCM (NMR measurements)

Solutions of dienes **6** and **8** (0.08 mmol) in degassed CD_2Cl_2 0.75 mL was placed in NMR tube. Solutions of catalysts 1 mol% was freshly prepared and added to NMR tube in 0.05 mL of CD_2Cl_2. Progress of the reactions was monitored by means of NMR spectroscopy and their products were unambiguously identified by comparing with published NMR spectra available in literature.

Diethyl cyclopent-3-ene-1,1-dicarboxlate (7)

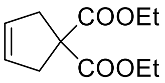

According to general procedure: substrate **6** (19.2 mg, 0.08 mmol) and catalyst **4a** (0.537 mg, 0.0008 mmol, 1 mol%) dissolved in CD_2Cl_2 (0.8 mL). Colourless oil. ^{1}H NMR (500 MHz, $CDCl_3$) δ = 1.24 (t, *J* = 7,1 Hz, 6H), 3.01 (s, 4H), 4.19 (q, *J* = 7,1 Hz, 4H), 5.61 (s, 2H) ppm. The spectrum correspond to this described in the literature.[2]

Catalyst	**4a**	**4b**	**4c**	**4d**	**4e**
TON	95	100	98	99	99

Diethyl 3-methylcyclopent-3-ene-1,1-dicarboxylate (9)

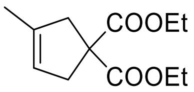

According to general procedure: substrate **8** (20.3 mg, 0.08 mmol) and catalyst **4a** (0.537 mg, 0.0008 mmol, 1 mol%) dissolved in CD_2Cl_2 (0.8 mL). Colourless oil. ^{1}H NMR (500 MHz, $CDCl_3$) δ = 1.24 (t, *J* = 7,0 Hz, 6H), 1.70 (d, *J* = 1,5 Hz, 3H), 2.93 (s, 2H), 2.96 (s, 2H), 4.19 (q, *J* = 7,0 Hz, 4H), 5.18 (d, *J* = 1,5 Hz, 1H) ppm. The spectrum correspond to this described in the literature.[2]

Comparative RCM experiments (GC measurements)

Reactions of dienes **10** and **12** in (toluene), [diene] = 0.02 M, 80 °C, 24 h; refer Figures 6-8 and Tables 3-4 were performed as follows. To a stirred solution of diene in toluene (5 mL) placed under argon in a Schlenk tube catalyst **4a-e** (1-5 mol %) and dodecane (used as an internal standard, 0.25–0.30 mmol) was added in a single portion at 40 °C and the reaction mixture was stirred for 24 h at the same temperature. Aliquots (0.25 mL), taken in regular intervals, were quenched immediately with ice-cold solution of ethyl-vinyl ether (0.25 mL, 2 M in CH_2Cl_2). Progress of the reactions was monitored by means of GC, using HP 5890 chromatograph with HP 5 column and their products were unambiguously identified by comparing with published NMR spectra available in literature.

3,4-Dimethyl-1-tosyl-2,5-dihydro-1*H*-pyrrole (11)

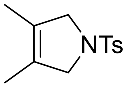

According to general procedure: substrate **10** (140 mg, 0.5 mmol), catalyst **4e** (21.4 mg, 0.025 mmol, 5 mol%), and dodecane (85.2 mg, 0.114 mL, 0.5 mmol) dissolved in toluene (5 mL). Colourless solid. ^{1}H NMR (400 MHz, $CDCl_3$) δ = 7.72 (d, 2H, J= 8.0 Hz), 7.32 (d, 2H, J= 8.0 Hz), 3.97 (s, 4H), 2.43 (s, 3H), 1.54 (s, 6H) ppm. The spectrum correspond to this described in the literature.[3]

Diethyl 3,4-dimethylcyclopent-3-ene-1,1-dicarboxylate (13)

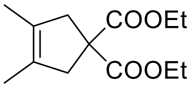

According to general procedure: substrate **12** (134 mg, 0.5 mmol), catalyst **4e** (21.4 mg, 0.025 mmol, 5 mol%), and dodecane (85.2 mg, 0.114 mL, 0.5 mmol) dissolved in toluene (5 mL). Yellowish oil. ^{1}H NMR (400 MHz, $CDCl_3$) δ = 4.20 (q, *J* = 7.12 Hz, 4H), 2.95 (m, 4H,), 2.90 (s, 2H), 1.61 (m, 6H), 1.26 (t, *J* = 7.12 Hz) ppm. The spectrum correspond to this described in the literature.[4]

Comparative ene-yne experiments (GC measurements)

Reactions of ene-ynes **14** and **16** were performed as follows. To a stirred solutions of ene-ynes in toluene (5 mL) C = 0.02 M placed under argon in a Schlenk tubes catalysts **4a-e** (1-5 mol %) and dodecane (used as an internal standard, 0.25–0.30 mmol) was added in a single portion at 40 °C and the reaction mixture was stirred for 24 h at the same temperature. Aliquots (0.25 mL), taken in regular intervals, were quenched immediately with ice-cold solution of ethyl-vinyl ether (0.25 mL, 2 M in CH_2Cl_2). Progress of the reactions was monitored by means of GC, using HP 5890 chromatograph with HP 5 column and their products were unambiguously identified by comparing with published NMR spectra available in literature.

2,2-Diphenyl-3-vinyl-2,5-dihydro-furan (15)

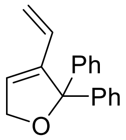

According to general procedure: substrate **14** (124 mg, 0.5 mmol), catalyst **4a** (3.36 mg, 0.005 mmol, 1 mol%), and dodecane (85.2 mg, 0.114 mL, 0.5 mmol) dissolved in toluene (5 mL). Brown oil. ^{1}H NMR (500 MHz, $CDCl_3$) δ = 4.11 (q, *J* = 7.1 Hz, 1H), 5.10 (dd, *J* = 11.2, 0.8 Hz, 1H), 5.31 (dd, *J* = 17.7, 0.8 Hz, 1H), 6.16–6.18 (m, 1H), 6.20–6.27 (m, 1H), 7.10–7.40 (m, 10H) ppm. The spectrum correspond to this described in the literature.[3]

4-Methyl-2,2-diphenyl-3-vinyl-2,5-dihydro-furan (17)

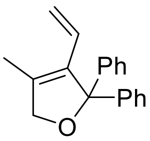

According to general procedure: substrate **16** (131 mg, 0.5 mmol), catalyst **4a** (16.8 mg, 0.025 mmol, 5 mol%), and dodecane (85.2 mg, 0.114 mL, 0.5 mmol) dissolved in toluene (5 mL). Brown oil. ^{1}H NMR (400 MHz, $CDCl_3$): δ = 7.37–26

(m, 10H), 6.24 (dd, J = 17.7, 11.7 Hz, 1H), 5.14 (d, J = 11.7 Hz), 5.02 (d, J = 17.7 Hz, 1H), 4.68 (s, 2H), 1.90 (s, 3H) ppm. The spectrum correspond to this described in the literature.[4]

X-Ray crystallography

For the **3b-3e** samples, the intensities of the diffracted X-ray radiation reflections were measured on a KM4CCD 4-axis diffractometer with graphite-monochromated Mo-Kα radiation (λ = 0.71073Å) and equipped with an Oxford Cryosystems nitrogen gas-flow apparatus. The data were corrected for Lorentz and polarization effects. Data reduction and analysis were carried out with the Oxford Diffraction Ltd. suite of programs. The crystals were mounted on Mounted CryoLoop with a droplet of the Pantone-N oil and immediately cooled.
The data collection and processing statistics are reported in Table 1. The structures were solved by direct methods using the SHELXS-97[5] program and refined with the SHELXL [1]. A multi-scan absorption correction was applied in the scaling procedure for all structures. After structure solution for **4d** and **4e**, it was found that 20% and 35% of the total cell volume was filled in with disordered solvent molecules. Their positions could not be modelled in terms of atomic sites. From this point on, residual peaks were removed and the solvent region was refined as a diffuse contribution without specific atom positions by using the PLATON[6] module SQUEEZE[7] which subtracts electron density from the void regions by appropriately modifying the diffraction intensities of the overall structure. An electron count over the solvent region provided an estimate for the number of solvent molecules removed from the cell. Applying this procedure led to an improvement in all refinement parameters and a minimization of residuals The experimental details for **4b**-**4e** are summarized in Table 1S.
Single-crystal X-ray measurements of the scattered intensities of reflections for **CuClPCy$_3$** samples carried out at 100K were performed using a Bruker AXS KAPPA-APEX II diffractometer equipped with a MoKα rotating anode X-ray source (λ = 0.71073 Å, 50.0 kV, 22.0 mA) monochromatized by multi-layer optics and APEX-II CCD detector and the Oxford Cryostream cooling device. The crystal was mounted on a Mounted CryoLoop with a droplet of Pantone-N oil and immediately cooled. Indexing, integration and initial scaling were performed with SAINT and SADABS software (Bruker AXS, Madison, U.S.A.). The crystals was positioned 50 mm from the CCD camera. 914 frames were measured at 0.5º intervals with a counting time of 10sec. The structures were solved by direct methods and refined using SHELXS-97. Multi-scan absorption corrections were applied in the scaling procedure. The experimental details for **CuClPCy$_3$** are summarized in Table 1S.
The refinement was based on F^2 for all reflections except those with negative intensities. Weighted R factors wR and all goodness-of-fit S values were based on F^2, whereas conventional R factors were based on the amplitudes, with F set to zero for negative F^2. The F_0^2> 2σ (F_0^2) criterion was applied only for R factors calculation was not relevant to the

choice of reflections for the refinement. The R factors based on F^2 are for all structures about twice as large as those based on *F*. The hydrogen atoms were located in the idealized geometrical positions, except the hydrogen in the solvent molecules. Scattering factors were taken from Tables 4.2.6.8 and 6.1.1.4 from the International Crystallographic Tables Vol.C.[8] All non-hydrogen atoms were refined anisotropically. All hydrogen atoms were placed in the idealized positions.

Table 1S. Experimental details for 4b-4e structures.

	**4b**	**4c**	**4d**	**4e**
**Crystal data**				
Chemical formula	$C_{37}H_{49}Cl_2N_3O_3Ru$	$C_{33}H_{39}Cl_2N_3O_3Ru$	$C_{45}H_{49}Cl_2N_3O_3Ru$	$C_{45}H_{53}Cl_2N_3O_3Ru$
M_r	755.76	697.64	851.84	855.87
Crystal system, space group	Monoclinic, *C*2/*c*	Monoclinic, $P2_1/c$	Triclinic, *P-1*	Monoclinic, $P2_1/n$
Temperature (K)	100	100	100	100
a, *b*, *c* (Å)	33.087 (2), 12.0080 (3), 22.1331 (13)	12.5391 (3), 14.1792 (4), 17.9276 (5)	11.3566 (6), 13.0025 (6), 15.0637 (6)	16.9146 (6), 29.5454 (15), 19.8773 (10)
β (°)	125.595 (9)	93.190 (3)	100.668 (4), 90.725 (4), 94.034 (4)	96.335 (4)
V (Å^3)	7150.6 (10)	3182.49 (15)	2179.76 (18)	9873.0 (8)
Z	8	4	2	8
Radiation type	Mo Kα, λ = 0.71073 Å	Mo Kα, λ = 0.71073 Å	Mo Kα, λ = 0.71073 Å	Mo Kα, λ = 0.71073 Å
μ (mm^{-1})	0.63	0.7	0.52	0.46
Crystal size (mm)	0.30 × 0.10 × 0.10	0.30 × 0.20 × 0.01	0.30 × 0.20 × 0.20	0.20 × 0.10 × 0.05
**Data collection**				
Diffractometer	KUMA4 CCD diffractometer	KUMA4 CCD diffractometer	KUMA4 CCD diffractometer	KUMA4 CCD diffractometer
Absorption correction	Multi-scan *CrysAlis PRO*, Agilent Technologies, Version 1.171.35.15 (release 03-08-2011 CrysAlis171 .NET) (compiled Aug 3 2011,13:03:54) Empirical absorption correction using spherical harmonics, implemented in SCALE3 ABSPACK scaling algorithm.	Multi-scan CrysAlis PRO, Agilent Technologies, Version 1.171.35.15 (release 03-08-2011 CrysAlis171 .NET) (compiled Aug 3 2011,13:03:54) Empirical absorption correction using spherical harmonics, implemented in SCALE3 ABSPACK scaling algorithm.	Multi-scan *CrysAlis PRO*, Agilent Technologies, Version 1.171.35.15 (release 03-08-2011 CrysAlis171 .NET) (compiled Aug 3 2011,13:03:54) Empirical absorption correction using spherical harmonics, implemented in SCALE3 ABSPACK scaling algorithm.	Multi-scan *CrysAlis PRO*, Agilent Technologies, Version 1.171.35.15 (release 03-08-2011 CrysAlis171 .NET) (compiled Aug 3 2011,13:03:54) Empirical absorption correction using spherical harmonics, implemented in SCALE3 ABSPACK scaling algorithm.
T_{min}, T_{max}	0.834, 0.940	0.818, 0.993	0.859, 0.903	0.913, 0.977
No. of measured, independent and observed [$I > 2\sigma(I)$] reflections	57053, 7247, 6418	30339, 5808, 4277	34449, 8274, 7324	18733, 18733, 10485
R_{int}	0.037	0.078	0.044	0
(sin θ/λ)$_{max}$ (Å^{-1})	0.625	0.602	0.61	0.61
**Refinement**				
$R[F^2 > 2\sigma(F^2)]$, $wR(F^2)$, S	0.046, 0.118, 1.37	0.041, 0.094, 1.07	0.036, 0.115, 1.16	0.112, 0.326, 1.11
No. of reflections	7247	5808	8274	18733
No. of parameters	420	389	488	973
No. of restraints	2	0	0	5

H-atom treatment	H-atom parameters constrained	H-atom parameters constrained	H-atom parameters constrained	H-atom parameters constrained
$\Delta\rho_{max}$, $\Delta\rho_{min}$ (e Å^{-3})	2.31, -2.91	0.46, -0.70	0.49, -0.96	1.71, -1.17

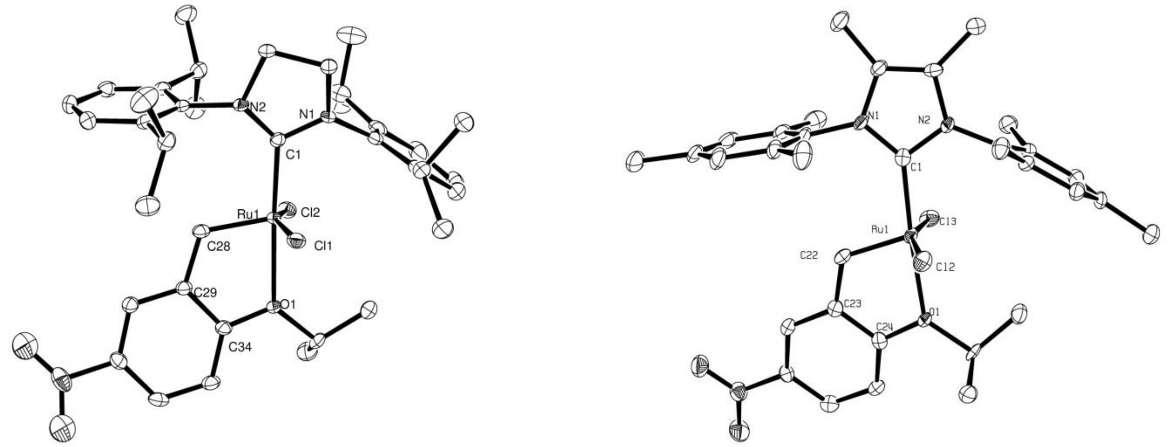

Figure 1S. Atomic Displacement Parameters (ADPs) and the labeling of atoms in **4b** and **4c**. Thermal ellipsoids are shown at the 50% probability level. Hydrogen atoms omitted for clarity.

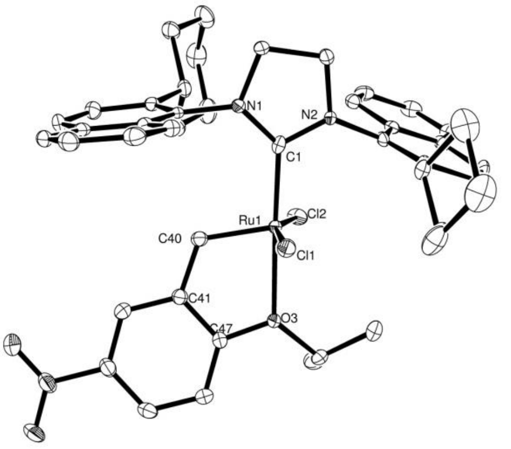

Figure 2S. Atomic Displacement Parameters (ADPs) and the labeling of atoms in **4d**. Thermal ellipsoids are shown at the 50% probability level. Hydrogen atoms omitted for clarity.

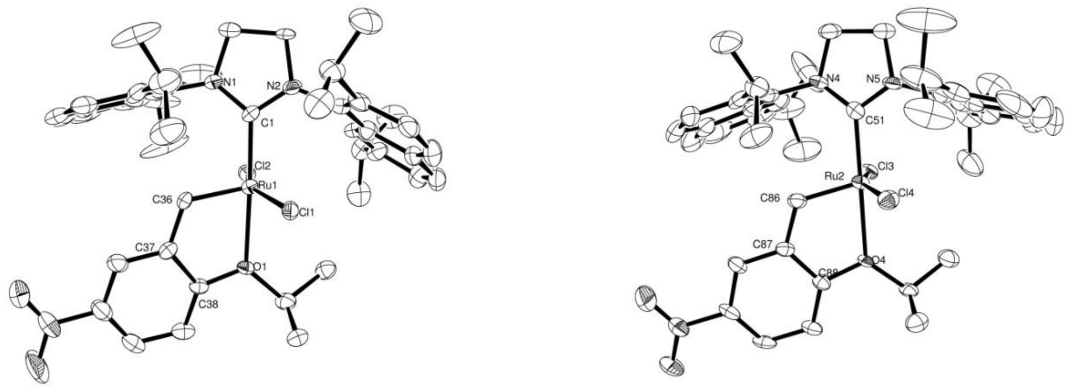

Figure 3S. Atomic Displacement Parameters (ADPs) and the labeling of atoms in **4e** for two molecules in asymmetric unit (**4e'** and **4e''**). Thermal ellipsoids are shown at the 50% probability level. Hydrogen atoms omitted for clarity.

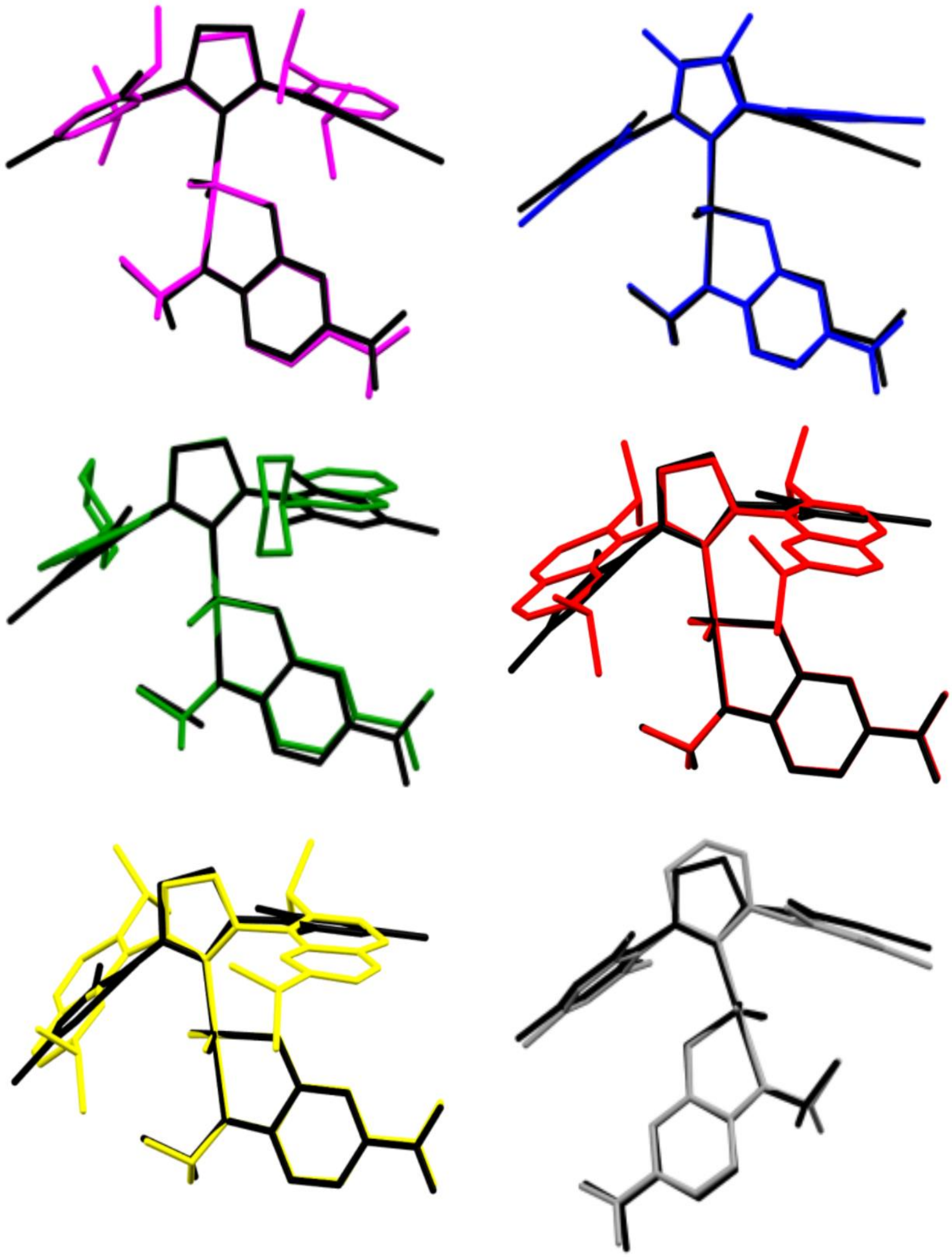

Figure 4S. Overlay of molecules from the **4a** structure (black) with the **4b** (magenta), **4c** structure (blue), **4d** structure (green), **4f** structure (grey), **4e'** structure (red) and **4e''** (yellow) and **4g** (grey).

The **CuClCy$_3$** molecules (Figure 5S) crystallize in the triclinic P-1 space group with half of the molecule in the asymmetric unit. The crystal structure of the complex was previously determined.[9] Each copper(I) atom is in trigonal coordination to a tricyclohexylphosphine ligand and two bridging chloride ligands. The bond distance between Cu-Cl1, Cu-Cl1^i and Cu-P1 is 2.3239(7) Å, 2.2931(5) Å and 2.1853(6), respectively. The Cu1^i-Cl1^i-Cu1 angle between is 83.57(5)° making the central ring a rhomboid. The *i* superscript stands for the symmetry operations 1-x, -y, -z.

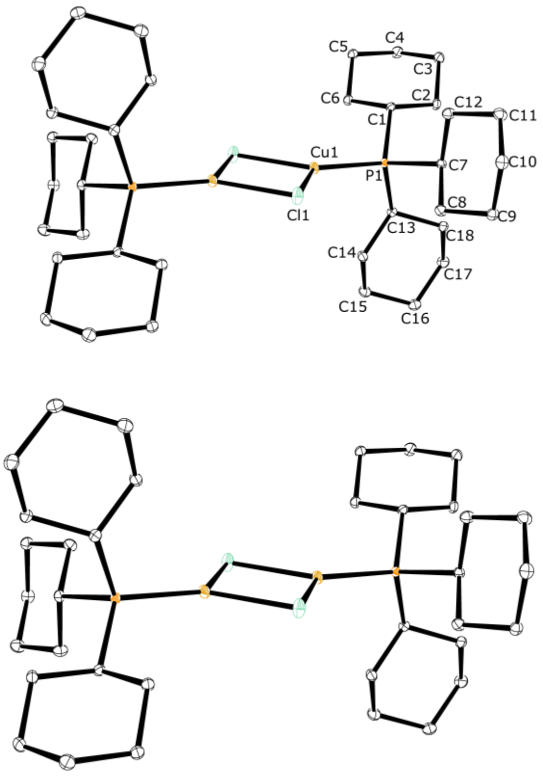

Figure 5S. Atomic Displacement Parameters (ADPs) and the labeling of atoms in **CuClCy₃**. Thermal ellipsoids are shown at the 50% probability level. Hydrogen atoms omitted for clarity.

Table 2S. Experimental details for the $CuClPCy_3$ measurement.

	**$CuClPCy_3$**
**Crystal data**	
Chemical formula	$C_{36}H_{66}Cl_2Cu_2P_2$
M_r	758.86
Crystal system, space group	Triclinic, $P\bar{1}$
Temperature (K)	100
a, *b*, *c* (Å)	8.3261 (6), 9.1977 (6), 13.2086 (9)
α, β, γ (°)	100.199 (2), 90.976 (2), 113.213 (2)
V (Å^3)	910.73 (11)
Z	1
Radiation type	Mo *K*α
μ (mm^{-1})	1.43
Crystal size (mm)	0.3 × 0.3 × 0.2
**Data collection**	
Diffractometer	Bruker Apex2 Ultra
Absorption correction	Multi-scan<br>*SADABS2008*/1 - Bruker Nonius area detector scaling and absorption

	correction
T_{min}, T_{max}	0.92, 0.98
No. of measured, independent and observed [$I > 2\sigma(I)$] reflections	7559, 3870, 3420
R_{int}	0.022
$(\sin \theta/\lambda)_{max}$ (Å^{-1})	0.642
Refinement	
$R[F^2 > 2\sigma(F^2)]$, $wR(F^2)$, S	0.032, 0.085, 1.06
No. of reflections	3870
No. of parameters	190
H-atom treatment	H-atom parameters constrained
$\Delta\rho_{max}$, $\Delta\rho_{min}$ (e Å^{-3})	0.55, -0.26

Computer programs: *SHELXL97* (Sheldrick, 1997).

NMR spectra

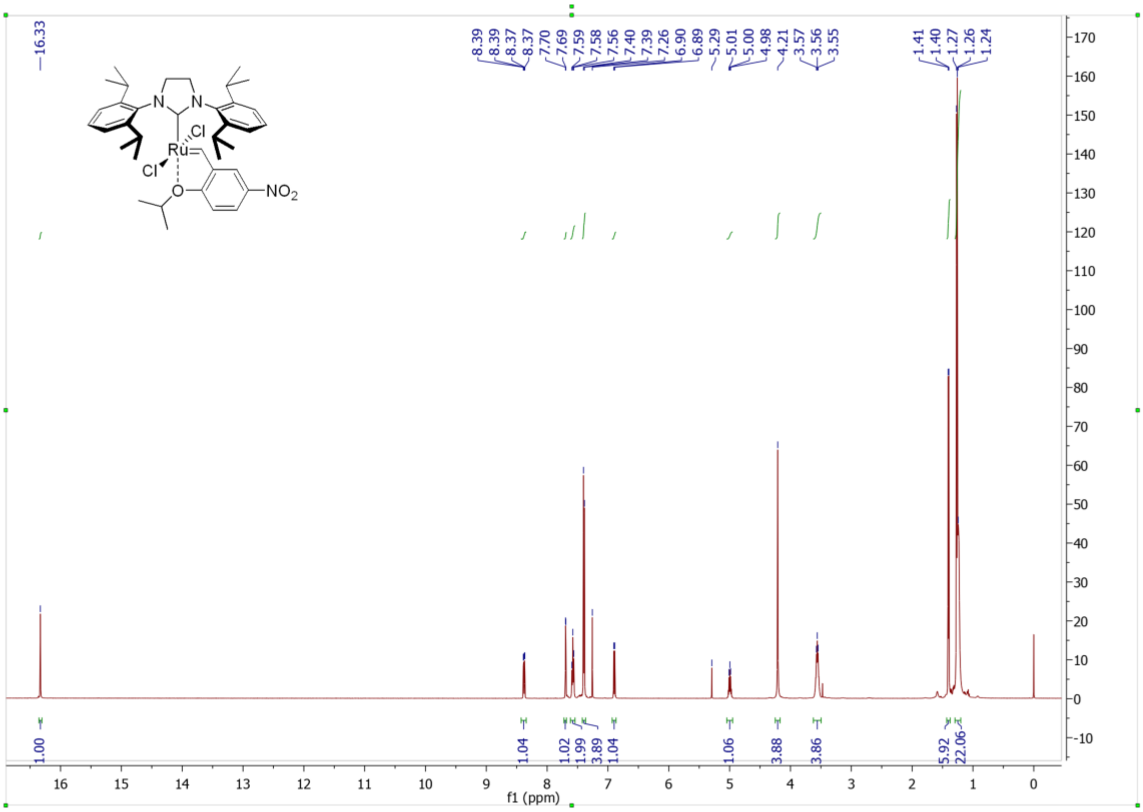
16.33
8.39 8.39 8.37 8.37 7.70 7.69 7.59 7.58 7.56 7.40 7.39 7.26 6.90 6.89 5.29 5.01 5.00 4.98 4.21 3.57 3.56 3.55
1.41 1.40 1.27 1.26 1.24
NO2
Cl
Ru
N
O
1.00
1.04
1.02
1.99
3.89
1.04
1.06
3.88
3.86
5.92
22.06
f1 (ppm)

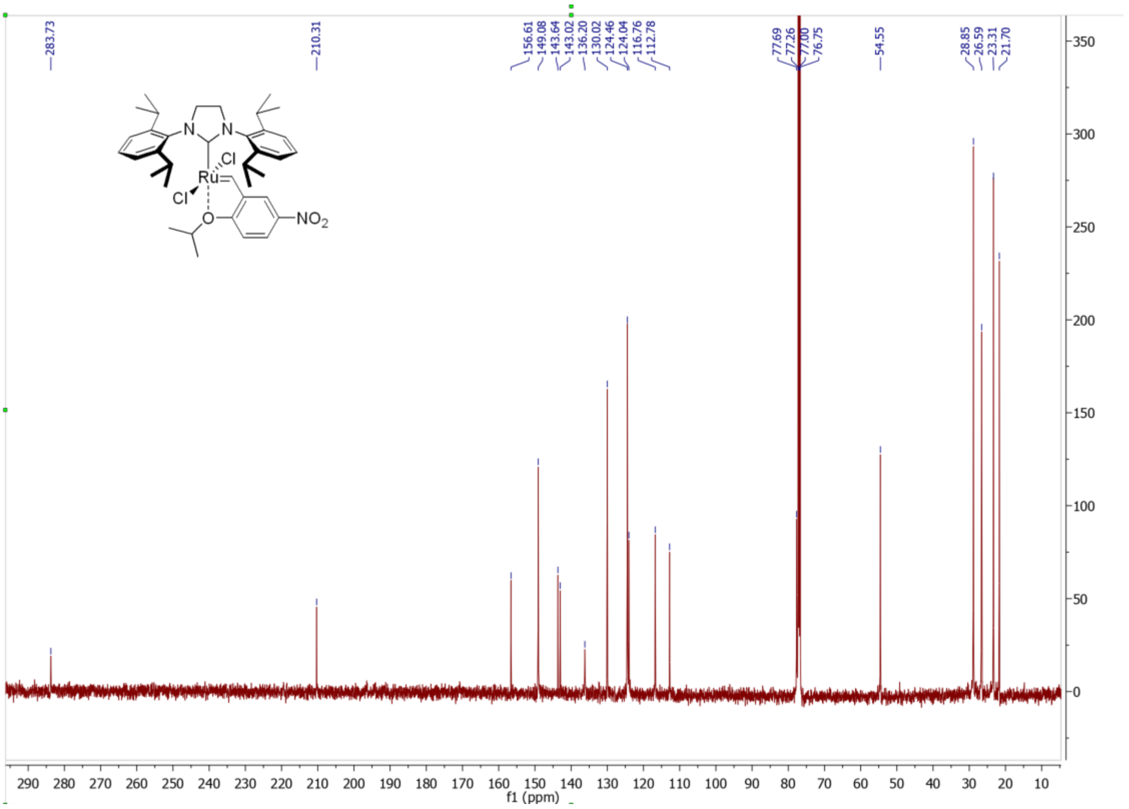
283.73
210.31
156.61 149.08 143.64 143.02 136.20 130.02 124.46 124.04 116.76 112.78
77.69 77.26 77.00 76.75
54.55
28.85 26.59 23.31 21.70
NO2
Cl
Ru
N
O
f1 (ppm)

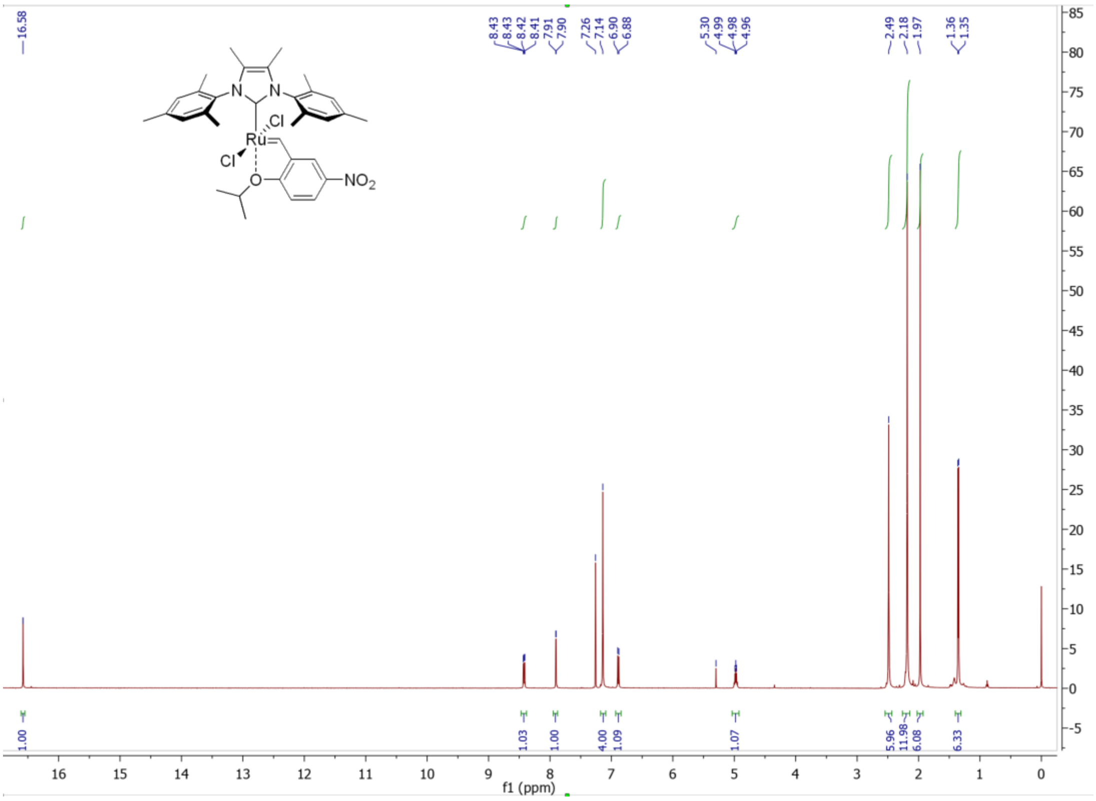
16.58
8.43 8.43 8.42 8.41 7.91 7.90 7.26 7.14 6.90 6.88
5.30 4.99 4.98 4.96
2.49 2.18 1.97 1.36 1.35
1.00 1.03 1.00 4.00 1.09 1.07 5.96 11.98 6.08 6.33
NO2
f1 (ppm)

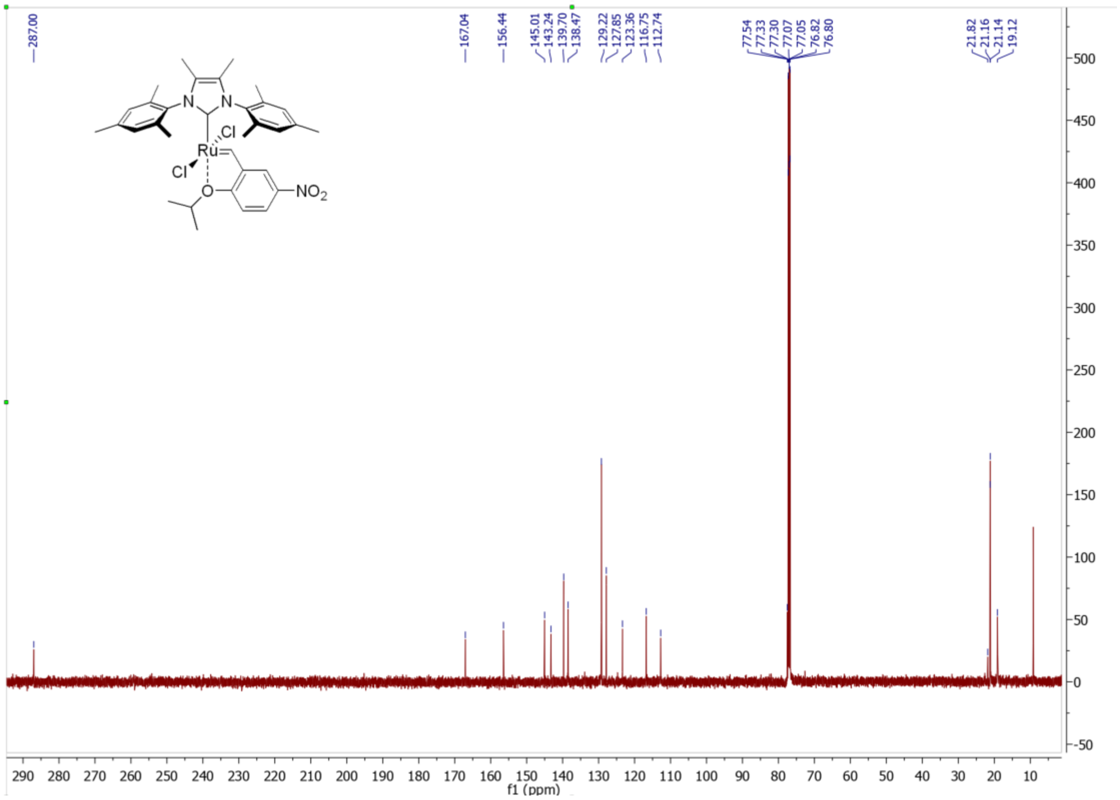
287.00
167.04 156.44 145.01 143.24 139.70 138.47 129.22 127.85 123.36 116.75 112.74
77.54 77.33 77.30 77.07 77.05 76.82 76.80
21.82 21.16 21.14 19.12
NO2
f1 (ppm)

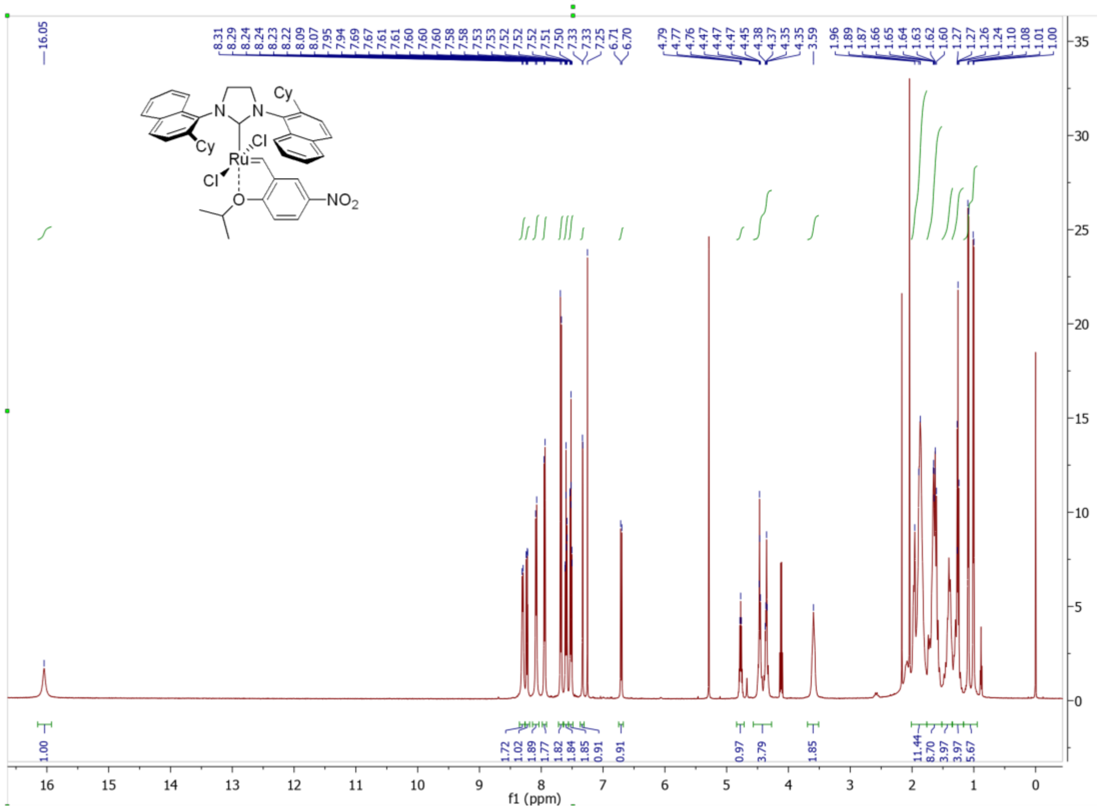
16.05
8.31
8.29
8.24
8.24
8.23
8.22
8.09
8.07
7.95
7.94
7.69
7.67
7.61
7.61
7.60
7.60
7.58
7.58
7.53
7.53
7.52
7.52
7.52
7.51
7.50
7.33
7.33
7.25
6.71
6.70
4.79
4.77
4.76
4.47
4.47
4.47
4.45
4.38
4.37
4.35
4.35
3.59
1.96
1.89
1.87
1.66
1.65
1.64
1.63
1.62
1.60
1.27
1.27
1.26
1.24
1.10
1.08
1.01
1.00
Cy
N
N
Cl
Cy
Ru
Cl
O
NO2
1.00
1.72
1.02
1.89
1.77
1.82
1.84
1.85
0.91
0.91
0.97
3.79
1.85
11.44
8.70
3.97
3.97
5.67
f1 (ppm)

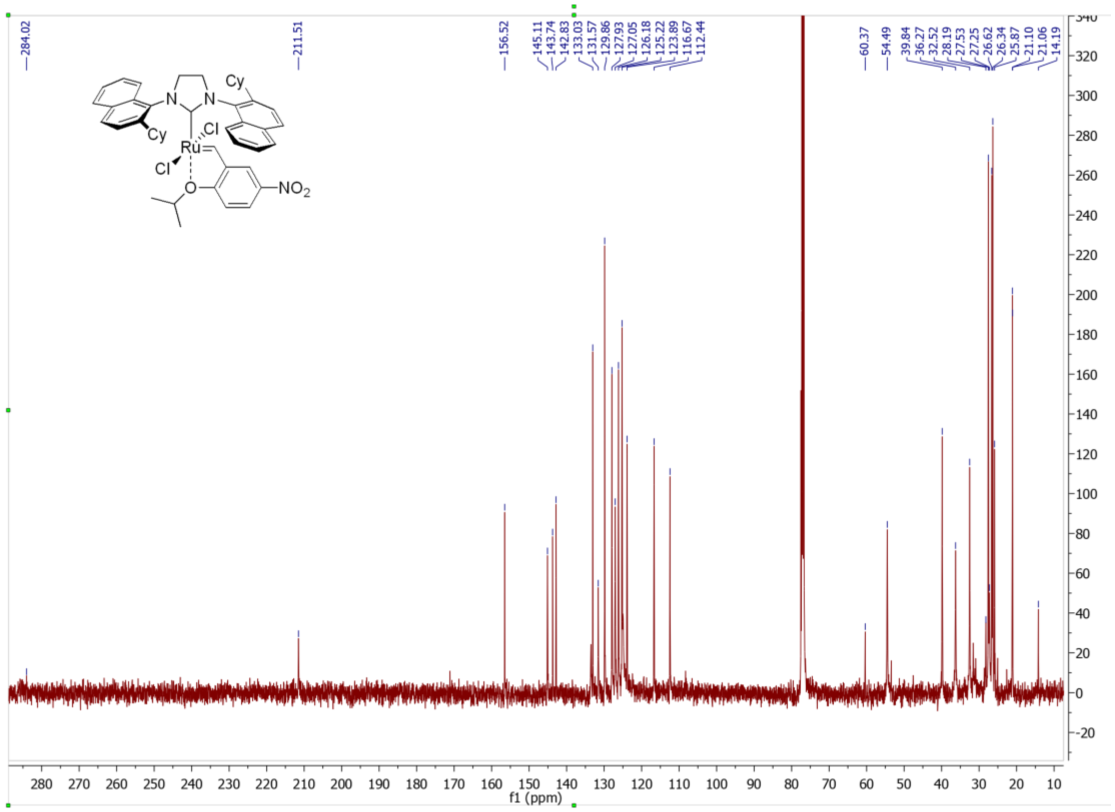
284.02
211.51
156.52
145.11
143.74
142.83
133.03
131.57
129.86
127.93
127.05
126.18
125.22
123.89
116.67
112.44
60.37
54.49
39.84
36.27
32.52
28.19
27.53
27.25
26.62
26.34
25.87
21.10
21.06
14.19
Cy
N
N
Cl
Cy
Ru
Cl
O
NO2
f1 (ppm)

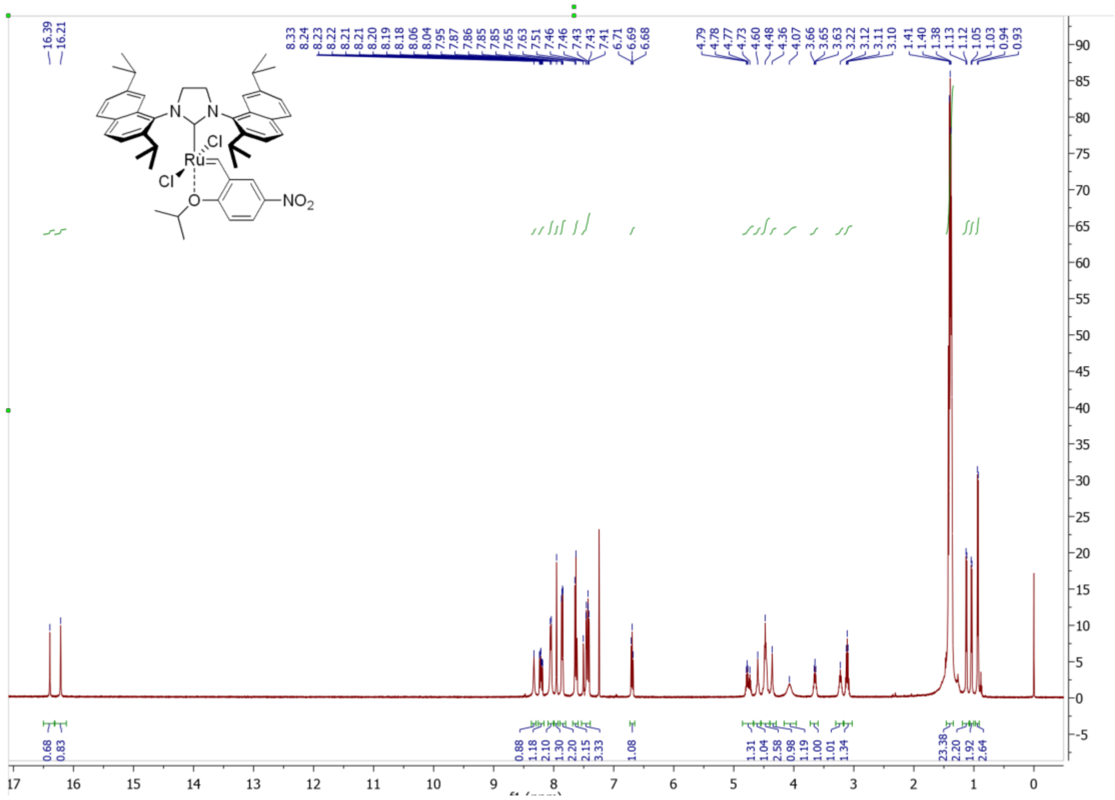
16.39
16.21
8.33 8.24 8.23 8.22 8.21 8.21 8.20 8.19 8.18 8.06 8.04 7.95 7.87 7.86 7.85 7.85 7.65 7.63 7.51 7.46 7.46 7.43 7.43 7.41 6.71 6.69 6.68
4.79 4.78 4.77 4.73 4.60 4.48 4.36 4.07 3.66 3.65 3.63 3.22 3.12 3.11 3.10 1.41 1.40 1.38 1.13 1.12 1.05 1.03 0.94 0.93
N
N
Ru
Cl
Cl
O
NO_2
0.68 0.83
0.88 1.18 2.10 1.30 2.20 2.15 3.33
1.08
1.31 1.04 2.58 0.98 1.19 1.00 1.01 1.34
23.38 2.20 1.92 2.64
17 16 15 14 13 12 11 10 9 8 7 6 5 4 3 2 1 0

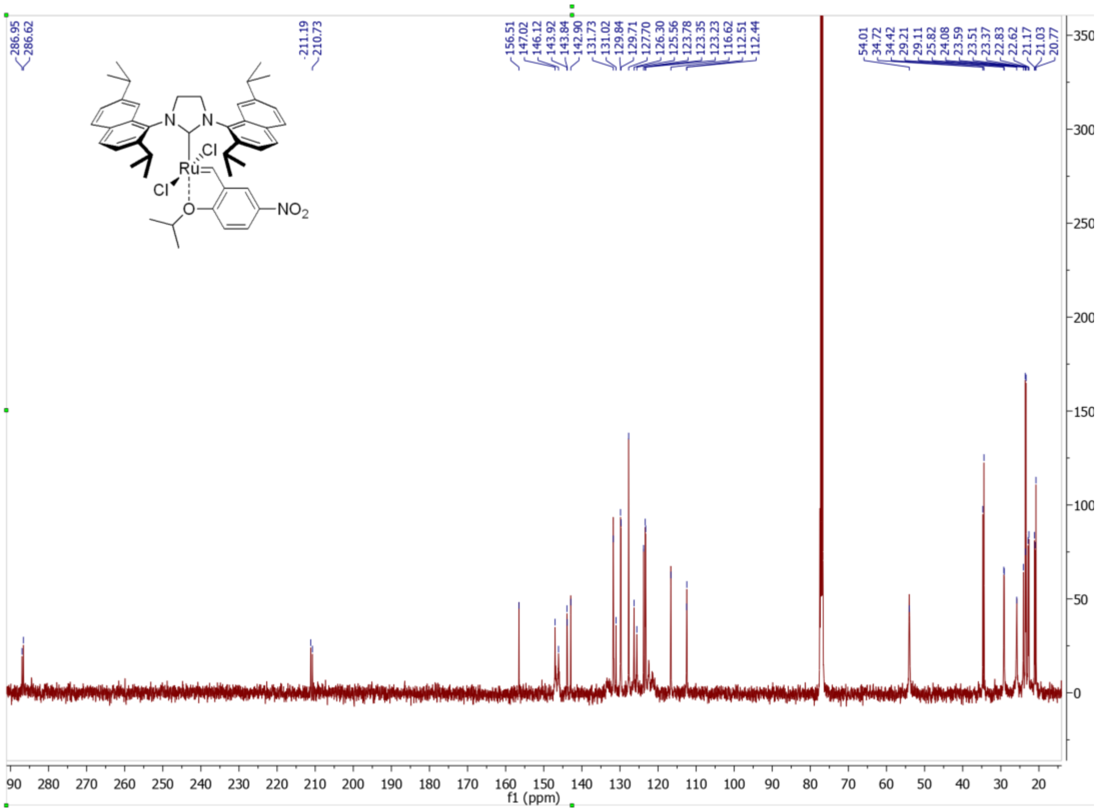
286.95
286.62
211.19
210.73
156.51 147.02 146.12 143.92 143.84 142.90 131.73 131.02 129.84 129.71 127.70 126.30 125.56 123.78 123.35 123.23 116.62 112.51 112.44
54.01 34.72 34.42 29.21 29.11 25.82 24.08 23.59 23.51 23.37 22.83 22.62 21.17 21.03 20.77
N
N
Ru
Cl
Cl
O
NO_2
290 280 270 260 250 240 230 220 210 200 190 180 170 160 150 140 130 120 110 100 90 80 70 60 50 40 30 20
f1 (ppm)

References

[1] a) Y. Kobayashi, S. Inukai, N. Kondo, T. Watanabe, Y. Sugiyama, H. Hamamoto, T. Shioiri, M. Matsugi, *Tetrahedron Lett*. **2015**, *56*, 1363-1366; b) J. M. Berlin, K. Campbell, T. Ritter, T. W. Funk, A. Chlenov, R. H. Grubbs, *Org. Lett*. **2007**, *9*, 1339-1342; c) H. Clavier, S. P. Nolan, *Chem. Eur. J*. **2007**, *13*, 8029-8036; d) T. E. Schmid, X. Bantreil, C. A. Citadelle, A. M. Z. Slawin, C. S. J. Cazin, *Chem. Commun*. **2011**, *47*, 7060-7062.

[2] D. F. Taber, K. J. Frankowski, *The Journal of Organic Chemistry* **2003**, *68*, 6047-6048.

[3] H. Clavier, S. P. Nolan, *Chemistry – A European Journal* **2007**, *13*, 8029-8036.

[4] J. Wappel, C. A. Urbina-Blanco, M. Abbas, J. H. Albering, R. Saf, S. P. Nolan, C. Slugovc, *Beilstein Journal of Organic Chemistry* **2010**, *6*, 1091-1098.

[5] G. M. Sheldrick, *Acta Cryst. A* **2008**, *64*, 112–122.

[6] A. L. Spek, *Acta Cryst. D* **2009**, *65*, 148–155.

[7] A. L. Spek, *Acta Cryst C,* **2015**, *71*, 9–18.

[8] A. J. C. Wilson, *International Tables for Crystallography*, Kluwer: Dordrecht, **1992**.

[9] M. R. Churchill, F. J. Rotella, *Inorg. Chem.* **1979**, *18*, 166–171.
